# Nonreciprocal forces enable cold-to-hot heat transfer between nanoparticles

**DOI:** 10.1038/s41598-023-31583-y

**Published:** 2023-03-18

**Authors:** Sarah A. M. Loos, Saeed Arabha, Ali Rajabpour, Ali Hassanali, Édgar Roldán

**Affiliations:** 1 Department of Applied Mathematics and Theoretical Physics, University of Cambridge, Wilberforce Road, Cambridge, CB3 0WA UK; 2 ICTP – International Centre for Theoretical Physics, Strada Costiera, 11, 34151 Trieste, Italy; 3 Department of Mechanical Engineering, Lassonde School of Engineering, York University, Toronto, Canada; 4 Advanced Simulation and Computing Laboratory (ASCL), Imam Khomeini International University, Qazvin, Iran; 5 School of Nano Science, Institute for Research in Fundamental Sciences (IPM), Tehran, Iran

**Keywords:** Statistical physics, thermodynamics and nonlinear dynamics, Thermodynamics, Nanoscale devices

## Abstract

We study the heat transfer between two nanoparticles held at different temperatures that interact through nonreciprocal forces, by combining molecular dynamics simulations with stochastic thermodynamics. Our simulations reveal that it is possible to construct nano refrigerators that generate a net heat transfer from a cold to a hot reservoir at the expense of power exerted by the nonreciprocal forces. Applying concepts from stochastic thermodynamics to a minimal underdamped Langevin model, we derive exact analytical expressions predictions for the fluctuations of work, heat, and efficiency, which reproduce thermodynamic quantities extracted from the molecular dynamics simulations. The theory only involves a single unknown parameter, namely an effective friction coefficient, which we estimate fitting the results of the molecular dynamics simulation to our theoretical predictions. Using this framework, we also establish design principles which identify the minimal amount of entropy production that is needed to achieve a certain amount of uncertainty in the power fluctuations of our nano refrigerator. Taken together, our results shed light on how the direction and fluctuations of heat flows in natural and artificial nano machines can be accurately quantified and controlled by using nonreciprocal forces.

## Introduction

Experimental techniques in single-molecule optical trapping and biophysics allow to extract real-time information of the state of a nanosystem with exquisite precision^1–3^. Such information is commonly used to infer both thermodynamical and dynamical properties through data-analysis techniques. Alongside, as inspired by Maxwell’s demon thought experiment, information acquired from a nanosystem can be delivered into work by executing feedback-control protocols^4–8^. In parallel to experimental progress, the development of stochastic thermodynamics (ST) over the last two decades provides a robust theoretical framework to describe accurately information-to-work transduction that takes into account nanoscale fluctuations^9–13^. Combining stochastic thermodynamics and feedback-cooling techniques has attracted attention towards refrigerating capabilities of small systems under nonequilibrium conditions^14,15^.

An important step to optimize the design of microscopic refrigerators is to bridge the gap between theoretical proposals and experiments through the powerful method of all-atoms simulations. Nonequilibrium Molecular Dynamics (MD) studies provide a suitable platform for the study of heat transfer and fluctuations at the nanoscale^16–20^. However, little is known yet about the design and performance of information demons at the atomic scale. In particular, are there generic principles that constrain the forces needed to ensure a prescribed value for the heat transfer between two thermal baths interacting through nanoscopic objects? Is it possible to accurately control the net heat transfer between nanoparticles and their respective fluctuations by *only* applying non-conservative forces, i.e. forces that do not derive from a potential?

Among the broad class of non-conservative forces, nonreciprocal interactions (i.e. forces that violate Newton’s third law “actio=reactio”) have recently emerged as a topic of lively interest in statistical physics^21–25^, revealing nontrivial physical consequences for the dynamical, mechanical and thermodynamic properties of many-body systems. For example, they introduce ‘odd elasticity’ in solids and soft crystals^26,27^ or lead to the formation of travelling waves in binary fluid mixtures^21–23^. In stochastic thermodynamics, recent research has revealed the potential of nonreciprocal forces in the design of artificial nano machines with efficient energetic performance^28^. Inspired by these recent findings, herein we design atomistic MD simulations of trapped nanoparticles immersed in thermal baths at different temperatures that interact through non-conservative, linear forces and that are nonreciprocal. A similar setup was realized experimentally very recently using optical fields^29^. Here, we use nonreciprocal interactions to construct a *nano refrigerator* which under certain conditions, achieves a steady, net heat flow from the cold to the hot bath. Interestingly, we show that although the value of this net cold-to-hot heat flow does not fulfill Fourier’s law for thermal conduction, it is nonetheless in agreement with recent theoretical predictions from ST. A key advantage of our nano refrigerator design, relies on its simplicity as it only requires the usage of nonreciprocal forces acting on each of the nanoparticles. This represents a simplification with respect to previous approaches where heat flows from hot to cold could be achieved using velocity-dependent feedback^30^ or memory registers^31,32^ as in Maxwell’s demons, or using nonlinear forces in athermal environments^33^.

Our work establishes theoretical design principles that ensure a prescribed net heat flux in our MD simulations that could be exported to realistic experimental scenarios with trapped nanoparticles^4,34–37^. We also test fundamental principles governing the fluctuations of work and the coefficient of performance (COP), some of which follow from recently-established thermodynamic uncertainty relations tested here with realistic atomistic simulations of nanoparticles^38,39^. These results push forward the synergistic combination of ST and MD beyond the application of fluctuation theorems in e.g. estimating free energies ^40,41^. In particular, our simulations made with parameters for realistic materials are a first step towards the engineered design of nanoparticle-based refrigerators powered by thermal fluctuations.

## Results

**Figure 1:**
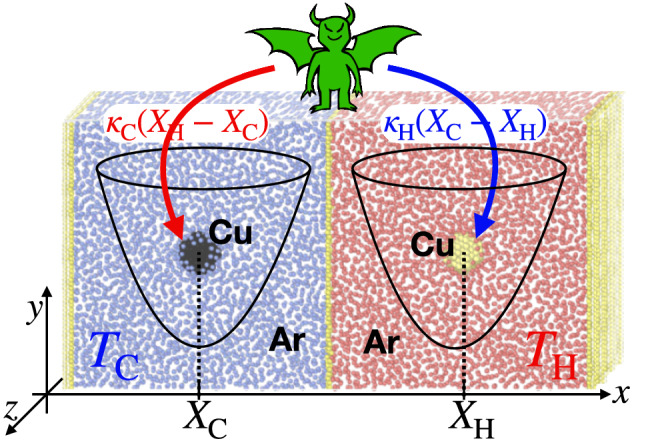
Sketch of the molecular dynamics (MD) simulation setup. Two copper nanoparticles (black and yellow spheres) each consisting of 186 atoms, are immersed in two Argon baths at different temperatures $T_{\mathrm{C}}=100 \, \mathrm{K}$ and $T_{\mathrm{H}}=120 \, \mathrm{K}$. The nanoparticles are trapped with two static three-dimensional harmonic potentials (see black line for an illustration). A demon-like controller (green) exerts additional nonreciprocal forces to the two nanoparticles as follows. The demon measures the nanoparticles’ positions $X_{\mathrm{C}},X_{\mathrm{H}}$ and exerts the forces $\kappa _{\mathrm{C}}(X_{\mathrm{H}}-X_{\mathrm{C}})$ to the nanoparticle in the cold bath and $\kappa _{\mathrm{H}}(X_{\mathrm{C}}-X_{\mathrm{H}})$ to the nanoparticle in the hot bath, where in general $\kappa _{\mathrm{H}}\ne \kappa _{\mathrm{C}}$ rendering the demon forces nonreciprocal.

### Nano demon setup and MD simulations

Constructing an atomistic MD simulation that allows us to violate Newton’s third law and furthermore realize a nonreciprocal nano refrigerator, requires a highly unconventional setup in nonequilibrium MD studies. Specifically, we simulate two independent Argon baths that are kept thermostatted at different temperatures ($T_{\mathrm{C}}=100$ K and $T_{\mathrm{H}}=120$ K). The cold and hot bath are in turn separated by a hard wall made of immobile copper particles (see Fig. 1). Within each bath, we immerse a copper-based spherical nanoparticle of radius $1.4 \mathrm{nm}$. External nonreciprocal forces (sketched as a green demon in Fig. 1) are applied on the center of each nanoparticle through two forces, $\kappa _{\mathrm{C}}(X_{\mathrm{H}}{-X_{\mathrm{C}})}$ and $\kappa _{\mathrm{H}}(X_{\mathrm{C}}{-X_{\mathrm{H}})}$ on the nanoparticles immersed in the cold and hot baths respectively. Here $X_{\mathrm{H}}$ and $X_{\mathrm{C}}$ denote respectively the center-of-mass position of the particle in the hot and the particle in the cold bath, respectively. Such a setup could, in principle, be realized with the help of an external control scheme (e.g., using optical feedback) similar to previous studies^24,42^. When $\kappa _{\mathrm{C}}\ne \kappa _{\mathrm{H}}$, the introduced force is nonreciprocal, since the *actio=reactio* principle: $\kappa _{\mathrm{C}}(X_{\mathrm{H}}{-X_{\mathrm{C}})} {=} - \kappa _{\mathrm{H}}(X_{\mathrm{C}}{-X_{\mathrm{H}})}$ is satisfied only when $\kappa _{\mathrm{C}}= \kappa _{\mathrm{H}}$. As we will see shortly, this nonreciprocal coupling can lead for specific values $\kappa _{\mathrm{C}}/\kappa _{\mathrm{H}}$ to a heat flow from the cold to the hot bath. We have further constrained the particle positions by introducing harmonic potentials (with stiffness $\kappa$ ). The traps prevent the particles from hitting the walls, but are, in principle, not needed to construct the nano refrigerator, (i.e., we could also set $\kappa =0$), as is evident from our analytical results introduced in the following. In all our MD simulations, we set the stiffness of the traps to the value ${\kappa }=1\,\mathrm {eV/(nm)^2} =1.16 k_{\mathrm{B}}T_{\mathrm{C}}/$Å$^2$. We further fix $\kappa _{\mathrm{C}}=10 \kappa$ and vary $\kappa _{\mathrm{H}}$.

### Heat transfer from MD simulations

Our MD setup gives us direct access to thermodynamic quantities allowing for quantitative measurements of the heat transfer between the nanoparticles and their respective solvent baths. Specifically, we determined the total amount of energy change by extracting both the potential and kinetic energy of all bath molecules as a function of time which gives the total heat transferred by the copper nanoparticles to both the cold and hot baths, denoted by $\mathrm{d}Q_{\mathrm{C}}$ and $\mathrm{d}Q_{\mathrm{H}}$ respectively. Integrating over the course of the MD simulation yields the $Q_{\mathrm{C}}$ and $Q_{\mathrm{H}}$, which directly encodes the stochastic heat dissipated by the nanoparticle into the cold and hot bath respectively. Note that we use the sign convention that ${Q}>0$ when net energy is dissipated from the nanoparticle to the bath and ${Q}<0$ when it is absorbed by the nanoparticle from the bath. We estimate the heat dissipation rate ${\dot{Q}}_{\mathrm{C}}$ and ${\dot{Q}}_{\mathrm{H}}$ from the slope of a linear regression on the cumulative $Q_{\mathrm{C}}$ and $Q_{\mathrm{H}}$ over time.

**Figure 2:**
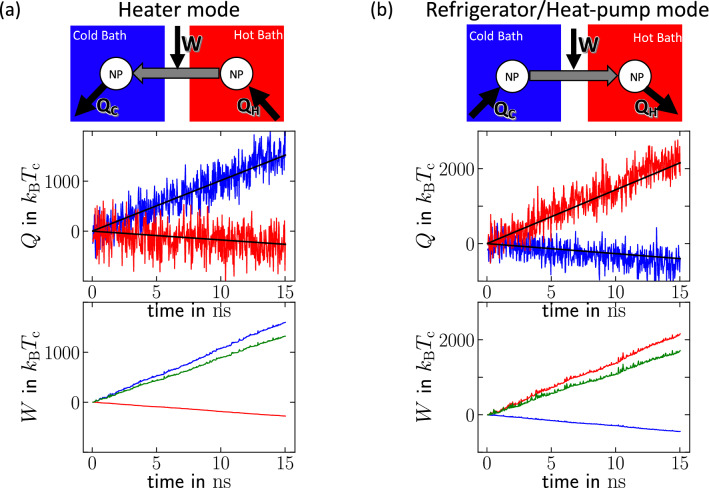
Heat flows *Q* measured in the MD simulations of the setup sketched in Fig. 1, for two different values of $\kappa _{\mathrm{H}}$. (**a**) *Heater Type-II*: for $\kappa _{\mathrm{H}} =0.17\kappa _{\mathrm{C}}$, heat flows from the hot bath to the nanoparticle in the right container and from the nanoparticle to the cold bath in the left container. (**b**) *Refrigerator/Heat-pump*: for $\kappa _{\mathrm{H}} =4.80\kappa _{\mathrm{C}}$, heat flows from the cold bath to the nanoparticle in the left container and from the nanoparticle to the hot bath in the right container. Here, heat flows in the reverse direction to the temperature gradient, i.e., it is extracted from the cold bath and released into the hot bath through the two-nanoparticle system. *Upper panels* of (**a**, **b**) show sketches of the flows of heat and work, respectively. *Middle panels* show the cumulative heat as function of time from the MD simulations, dissipated into the cold bath ($Q_{\mathrm{C}}$, blue line) and from the hot bath ($Q_{\mathrm{H}}$, red line). We use the convention $Q>0$ when heat flows from the nanoparticle into the bath and $Q<0$ when heat flows from the bath to the nanoparticle. Black lines show linear fits used to obtain the heat rates. *Lower panels* display the stochastic work exerted on the nanoparticle in the cold bath ($W_{\mathrm{C}}$, blue line), and on the nanoparticle in the hot bath ($W_{\mathrm{H}}$, red line), and the total work given by their sum ($W=W_{\mathrm{C}}+W_{\mathrm{H}}$, green line). The work is obtained from the MD simulations by measuring the center of mass positions of the nanoparticles, and evaluating the work using Eq. (8). Throughout, we used $\kappa _{\mathrm{C}}=10\kappa =11.6 \, k_{\mathrm{B}}T_{\mathrm{C}}/$Å$^2$, $T_{\mathrm{C}}= 100 \, \mathrm{K}$ and $T_{\mathrm{H}}=120 \, \mathrm{K}$.

With this protocol in hand, we begin by demonstrating how tuning the relative strength of $\kappa _{\mathrm{C}}$ and $\kappa _{\mathrm{H}}$ provides a microscopic mechanism to alter the direction of heat flow. Figure 2a illustrates $Q_{\mathrm{C}}$ and $Q_{\mathrm{H}}$ a situation where the effective coupling force on the hot particle is reduced as $\kappa _{\mathrm{H}}\ll \kappa _{\mathrm{C}}$. In this case, we observe the canonical situation, where the nanoparticle-duet behaves as a *heater*, i.e. heat flows from the hot to the cold bath. On the other hand, by introducing an effectively enhanced coupling force experienced by the nanoparticle in the hot bath, there is a striking effect where the direction of the heat flow changes as seen in Fig. 2b—heat is now pumped from the cold to the hot bath creating a molecular-scale refrigerator. The preceding results from the MD simulations provide a powerful proof-of-concept on how nonreciprocal forces applied on two nanoparticles embedded in a solvent bath, can in principle be used to change both the rate and direction of heat flow.

Intuitively, by increasing $\kappa _{\mathrm{H}}$, the demon tricks the hot particle into *“seeing”* that it was coupled to an even hotter particle, while the cold particle thinks it was coupled to an even colder particle, which results in a heat transfer from the cold to the hot bath. In the following, we will formulate and present a theory that rationalizes this intriguing phenomenon and makes a direct link between thermodynamic observables extracted from the MD simulations and stochastic thermodynamics.

### Stochastic model

We employ a mesoscopic stochastic model to describe the nonequilibrium dynamics of the position and momentum fluctuations of the two-nanoparticle system in their thermal environments. To this aim, we describe at a coarse-grained level the dynamics of the *x*-components of the positions and velocities of the center of mass of the nanoparticles, $X_{\mathrm{C}}$ and $X_{\mathrm{H}}$, by two coupled underdamped Langevin equations,

1 $$\begin{aligned} m {\ddot{X}}_{\mathrm{C}}+\gamma _{\mathrm{C}} {\dot{X}}_{\mathrm{C}}&= - {\kappa } X_{\mathrm{C}} + \kappa _{\mathrm{C}} {(X_{\mathrm{H}} -X_{\mathrm{C}})}+ \xi _{\mathrm{C}} \end{aligned}$$

2 $$\begin{aligned} m {\ddot{X}}_{\mathrm{H}}+\gamma _{\mathrm{H}} {\dot{X}}_{\mathrm{H}}&= -{\kappa }X_{\mathrm{H}} + \kappa _{\mathrm{H}}{(X_{\mathrm{C}} -X_{\mathrm{H}} )}+ \xi _{\mathrm{H}}. \end{aligned}$$

Here, *m* is the mass of each nanoparticle, and the coefficients $\kappa _{\mathrm{C}}$, $\kappa _{\mathrm{H}}$, $\kappa$ have been defined before. Note that the dynamics is independent of the actual distance between both nanoparticles, which has therefore been excluded from the equations of motions (1). The stochastic forces $\xi _{\mathrm{C}}$, $\xi _{\mathrm{H}}$ are independent Gaussian white noises with zero mean $\langle \xi _{\mathrm{C}}(t)\rangle =\langle \xi _{\mathrm{H}}(t)\rangle =0$ modeling the thermal noise exerted by the Argon bath that surrounds each nanoparticle. Their autocorrelation functions are $\langle \xi _j(t)\xi _l(t') \rangle =2 k_{\mathrm{B}}T_\mu \gamma _\mu \,\delta _{jl}\delta (t-t')$, where $j,l\in \{\mathrm{C,H}\}$ are indices denoting the hot or cold bath, $\delta _{jl}$ is Kroneckers’ delta, and $k_{\mathrm{B}}$ is Boltzmann’s constant. Here and in the following, $\langle . \rangle$ denote averages over many realizations of the noise. The averages obtained from the MD simulations are extracted from single trajectories of 15ns long, i.e. exceeding by three orders of magnitude the relaxation times of our system (see below). Furthermore, $\gamma _{\mathrm{C}}$ and $\gamma _{\mathrm{H}}$ are *effective* coefficients of the friction that each nanoparticle experiences in its respective environment. Despite its simplicity, the model (1) allows us to infer dynamical and thermodynamic properties of our molecular dynamics simulations, as we describe below.

An important ingredient for the theory are the effective friction coefficients $\gamma _{\mathrm{C}}$ and $\gamma _{\mathrm{H}}$, which emerge from the interaction of the nanoparticles with the baths’ particles^43^. To estimate $\gamma _{\mathrm{C}}$ and $\gamma _{\mathrm{H}}$, we run an equilibrium simulation in the absence of (nonreciprocal) interactions by taking $\kappa _{\mathrm{C}}=\kappa _{\mathrm{H}}=0$. In this limit, the position and velocity autocorrelation functions can be solved analytically (see Methods section). By fitting the autocorrelation functions obtained from the MD simulations to the analytical formulas, we extract the estimates $\gamma _{\mathrm{C}}\simeq \gamma _{\mathrm{H}} \sim 3.5 \times 10^{-12}$kg/s. This value is consistent with an independent estimate obtained from a *dragging experiment* simulation where a nanoparticle is pulled with a constant force along the *x* axis (see the Supplemental for further details), which yields an estimate of $\gamma _{\mathrm{C}} \approx \gamma _{\mathrm{H}} \approx 3\times 10^{-12}$kg/s. In the following and for further analyses, we use the estimate $\gamma = 3.5 \times 10^{-12}$kg/s for the friction of the two nanoparticles.

In the ensuing analysis, several timescales are relevant to characterize the dynamics of the particles at the mesoscopic level, following (1) and (2). Firstly, the momentum relaxation time extracted from our data $m/\gamma \approx 5$ ps is only one order of magnitude smaller than the relaxation time for the position in the trap $\gamma /{\kappa } \approx 25$ ps. These results lend credence to the validity of the underdamped description used in our approach, as the data acquisition timescale $\Delta t\sim 1$ps is below the momentum relaxation timescale.

### Stochastic energetics

**Figure 3:**
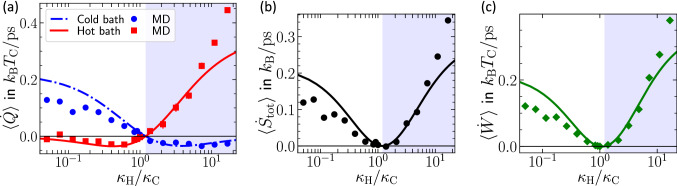
Comparison between thermodynamic fluxes obtained from MD simulations (symbols) and theoretical predictions from stochastic thermodynamics (lines). (**a**) The average rate of heat dissipation rate in each bath, (**b**) the average entropy production rate, and (**c**) the total power applied by the demon to the system, as a function of the coupling constant $\kappa _{\mathrm{H}}$. The heat rates in (**a**) are measured by accumulating the amount of energy extracted by the thermostats and linearly fitting the results to obtain the slope, see Fig. 2. The blue shaded area illustrates the parameter regime at which the system works as a refrigerator [given by the condition (6)]

We now develop and put to the test a theory for the energetics of the nanoparticle system setup in the light of stochastic thermodynamics^9^—a framework that enables to describe the fluctuations of heat and work of systems described by e.g. Langevin equations, such as Eq. (1). Applying the framework of stochastic thermodynamics to the model given by Eq. (1), and putting forward mathematical techniques introduced in^44–46^, we derive exact closed-form expressions for the expected value of the rate of heat dissipated into the cold and hot baths’ in the steady state, reading as,

3 $$\begin{aligned}{} & {} \langle {\dot{Q}}_{\mathrm{C}}\rangle =-k_{\mathrm{B}} \frac{ \displaystyle \kappa _{\mathrm{C}} (\kappa _{\mathrm{C}} T_{\mathrm{H}}-\kappa _{\mathrm{H}} T_{\mathrm{C}})}{\displaystyle (m/\gamma ) (\kappa _{\mathrm{C}} + \kappa _{\mathrm{H}})^2 /2+ \gamma (2\kappa +\kappa _{\mathrm{C}}+\kappa _{\mathrm{H}})}, \end{aligned}$$

4 $$\begin{aligned}{} & {} \langle {\dot{Q}}_{\mathrm{H}}\rangle =-k_{\mathrm{B}} \frac{\displaystyle - \kappa _{\mathrm{H}} (\kappa _{\mathrm{C}} T_{\mathrm{H}}-\kappa _{\mathrm{H}} T_{\mathrm{C}})}{\displaystyle (m/\gamma ) (\kappa _{\mathrm{C}} + \kappa _{\mathrm{H}})^2 /2+ \gamma (2\kappa +\kappa _{\mathrm{C}}+\kappa _{\mathrm{H}})}. \end{aligned}$$

To ensure that the dynamics of the nanoparticles is stable, we further find the necessary condition $\kappa _{\mathrm{H}} + \kappa _{\mathrm{C}} > -\kappa$ (see the Supplemental Material). Note that in the above formulas, the steady-state averages $\langle {\dot{Q}}_{\mathrm{C}}\rangle = \langle \mathrm{d}{Q}_{\mathrm{C}}/\mathrm{d}t\rangle$ and $\langle {\dot{Q}}_{\mathrm{H}}\rangle = \langle \mathrm{d}{Q}_{\mathrm{H}}/\mathrm{d}t\rangle$ are obtained using the definitions of stochastic heat used in stochastic thermodynamics (see Methods section), which are written in terms of the nanoparticles’ positions and velocities and thus not necessarily equal to the “direct” stochastic heat measured in the MD simulations from the energy fluctuations of the bath molecules.

Notably, Eqs. (3)–(4) predict a net heat transfer between the two baths that obeys Fourier’s law $\langle {\dot{Q}}_{\mathrm{C,H}}\rangle \propto (T_{\mathrm{H}}-T_{\mathrm{C}})$ only when the forces exerted by the demon are reciprocal. This result is consistent with the findings of experimental work with microparticles interacting solely through reciprocal forces. In particular, the Fourier law was tested using two optically-trapped microscopic particles held at different effective temperatures and coupled through hydrodynamic forces^35^. Moreover, our theory predicts that a net heat flow can be induced by three mechanisms: first, the existence of a temperature gradient, second, nonreciprocal coupling, or, third, a combination of both. As we show below, such a net heat flow is a signature of entropy production, with the latter being also accessible from our theory. In particular, Eqs. (3) and (4) allow for the prediction of a closed-form theoretical expression for the steady-state average rate of entropy production $\langle {\dot{S}}_{\mathrm{tot}}\rangle = \langle {\dot{Q}}_{\mathrm{H}}\rangle /T_{\mathrm{H}} +\langle {\dot{Q}}_{\mathrm{C}} \rangle /T_{\mathrm{C}}$^11^, yielding

5 $$\begin{aligned} \langle {\dot{S}}_{\mathrm{tot}} \rangle&= \frac{k_{\mathrm{B}}( \kappa _{\mathrm{C}}T_{\mathrm{H}} - \kappa _{\mathrm{H}} T_{\mathrm{C}})^2/(T_{\mathrm{C}}T_{\mathrm{H}})}{(m/\gamma ) (\kappa _{\mathrm{C}} +\kappa _{\mathrm{H}})^2/2 + \gamma (\kappa _{\mathrm{C}}+\kappa _{\mathrm{H}}+2\kappa )} . \end{aligned}$$

Note that $\langle {\dot{S}}_{\mathrm{tot}} \rangle \ge 0$ for any parameter values, in agreement with the second law of stochastic thermodynamics, with equality only for the choice $\kappa _{\mathrm{H}}/ \kappa _{\mathrm{C}}= T_{\mathrm{H}}/T_{\mathrm{C}}$ for the nonreciprocal coupling constants. Furthermore, from Eqs. (3) and (4) we can also extract the total power exerted by the demon on the nanoparticles, which is simply given by $\langle {\dot{W}}\rangle = \langle {\dot{Q}}_{\mathrm{C}}+{\dot{Q}}_{\mathrm{H}} \rangle$, as shown below.

To gain further insights, we compare in Fig. 3a our theoretical predictions from stochastic thermodynamics [Eqs. (3)–(4), lines] for the heat transfer evaluated directly over a collection of MD simulations (symbols) ran over a wide range of values for the demon coupling constants $\kappa _{\mathrm{H}}$ and $\kappa _{\mathrm{C}}$ spanning three orders of magnitude in $\kappa _{\mathrm{H}}/\kappa _{\mathrm{C}}$. Figure  3a reveals an excellent semi-quantitative agreement between the direct heat measurement in MD simulations with the stochastic theory over the parameter regime that we explore. Notably, we remark that our predictions are done without using any fitting parameter, i.e. we use in Eqs. (3) and (4) the actual parameter values of the MD simulation and the friction coefficient estimated from the equilibrium fluctuations described above. Importantly, the MD simulation results reveal that the system acts as a heater whenever $\kappa _{\mathrm{H}}/ \kappa _{\mathrm{C}}< T_{\mathrm{H}}/T_{\mathrm{C}}$ and as a refrigerator (and heat-pump) when

6 $$\begin{aligned} \kappa _{\mathrm{H}}/ \kappa _{\mathrm{C}}> T_{\mathrm{H}}/T_{\mathrm{C}}, \end{aligned}$$

a feature that is supported by our theory. This result rationalizes the findings in Fig. 2, which correspond to $\kappa _{\mathrm{H}}/ \kappa _{\mathrm{C}}=0.17$ (Fig. 2a) and $\kappa _{\mathrm{H}}/ \kappa _{\mathrm{C}}=4.8$ (Fig. 2b) which correspond respectively to the heater and refrigerator regimes as $T_{\mathrm{H}}/T_{\mathrm{C}}=1.2$.

We also compare in Fig. 3b the theoretical prediction (5) for steady-state rate of entropy production (line) with its value estimated from the MD simulations (symbols), with the latter evaluated by plugging in to $\langle {\dot{S}}_{\mathrm{tot}}\rangle = \langle {\dot{Q}}_{\mathrm{H}}\rangle /T_{\mathrm{H}} +\langle {\dot{Q}}_{\mathrm{C}} \rangle /T_{\mathrm{C}}$, the MD values of the heat flows in the thermostats divided by their respective temperatures.

The estimate for the rate of entropy production obtained from the MD measurements is in good agreement with the theoretical expression given by (5). Around the coupling values $\kappa _{\mathrm{C}} T_{\mathrm{H}} =\kappa _{\mathrm{H}} T_{\mathrm{C}}$ the heat flow and entropy production vanish, which we will refer to as a “pseudo equilibrium” point in the following. In the Supplemental Material, we show that in this case, detailed balance holds, i.e., all probability currents vanish, despite the presence of a temperature gradient in the system together with a nonreciprocal coupling.

### Power and performance

**Figure 4:**
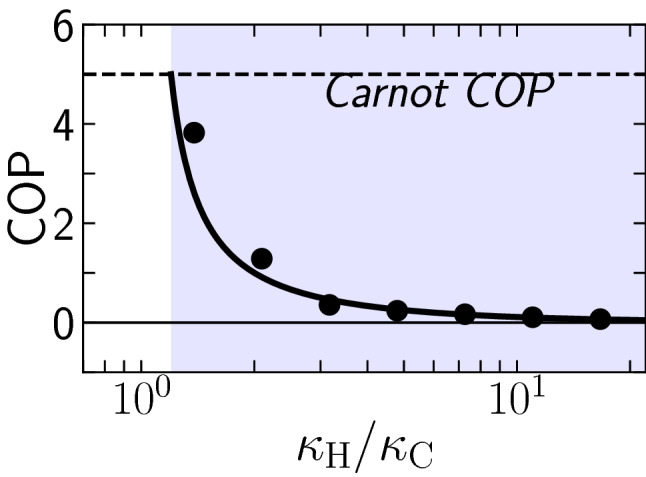
Average coefficient of performance as a function of $\kappa _{\mathrm{H}}$ in the refrigeration mode: results from MD simulations (symbols) and theoretical prediction from stochastic thermodynamics [(7), line]. The horizontal line indicates the Carnot bound for the coefficient of performance, reached at $\kappa _{\mathrm{H}}= \kappa _{\mathrm{C}} T_{\mathrm{H}}/T_{\mathrm{C}}$, and given by $\mathrm{{COP}} = T_{\mathrm{C}}/ (T_{\mathrm{H}} -T_{\mathrm{C}})$. The rest of the simulation parameters were set the same as in Fig. 2.

#### Performance of the nano refrigerator

In any refrigerator, getting the most heat from the temperature source is desirable by doing the least amount of work possible. Therefore, a suitable coefficient of performance (COP) is defined as the ratio of the heat taken from the low-temperature source to work done on the machine. A higher COP indicates a better economic performance of the system. In macroscopic systems, COP is usually in the range of $1-4$^47^. To quantify the net energetic performance of the nano refrigerator, we evaluate its coefficient of performance, defined by the average rate of heat that is extracted from the cold bath divided by the average total power inputted into the system, COP$=|\langle {\dot{Q}}_{\mathrm{C}}\rangle |/\langle {\dot{W}}\rangle$. From this definition and upon using our theoretical predictions for the heat transfer given by Eqs. (3) and (4), we predict that the COP follows

7 $$\begin{aligned} \mathrm{COP} \equiv \frac{{|\langle {\dot{Q}}_{\mathrm{C}}\rangle |}}{\langle {\dot{W}}\rangle } = \frac{{-\langle {\dot{Q}}_{\mathrm{C}}\rangle } }{\langle {\dot{Q}}_{\mathrm{H}}\rangle +\langle {\dot{Q}}_{\mathrm{C}}\rangle } = \frac{{\kappa _{\mathrm{C}}}}{{\kappa _{\mathrm{H}}}-{\kappa _{\mathrm{C}}}} . \end{aligned}$$

In (7), the second equality follows from using the first law of thermodynamics for stationary states which in this case reads $\langle {\dot{W}}\rangle -\langle {\dot{Q}}_{\mathrm{C}}\rangle -\langle {\dot{Q}}_{\mathrm{H}}\rangle =0$, whereas the third equality follows from Eqs. (3) and (4). Remarkably, our theoretical prediction for the COP depends solely on the nonreciprocal coupling parameters $\kappa _{\mathrm{H}}$ and $\kappa _{\mathrm{C}}$. Figure 4 reveals a good agreement between the value of the $\mathrm{COP}$ estimated from the MD simulations (symbols) and the prediction from our stochastic theory (7) (lines). Interestingly the agreement between simulation and theory is enhanced especially in far from equilibrium conditions, i.e. for large relative strengths of nonreciprocity $\kappa _{\mathrm{H}}/\kappa _{\mathrm{C}}\gg 1$. Close to the pseudo equilibrium $\kappa _{\mathrm{H}}/\kappa _{\mathrm{C}}=T_{\mathrm{H}}/T_{\mathrm{C}}$ point, the noise in the simulation results seems more pronounced. Towards this point, the theory predicts Carnot efficiency, which corresponds to a COP of $T_{\mathrm{C}}/(T_{\mathrm{H}}-T_{\mathrm{C}})=5$ in the present case.

#### Fluctuations of power and performance

We have shown so far that, when $\kappa _{\mathrm{H}}/\kappa _{\mathrm{C}}>T_{\mathrm{H}}/T_{\mathrm{C}}$, the nanoparticle system behaves like a refrigerator *on average*, i.e. its steady-state average fluxes obey $\langle {\dot{Q}}_{\mathrm{C}}\rangle >0$, $\langle {\dot{Q}}_{\mathrm{H}}\rangle <0$ and $\langle {\dot{W}}\rangle >0$. Due to thermal fluctuations, the two-nanoparticle system can give rise to transient values of the fluxes that do not obey the refrigerator constraints (e.g. transient values ${\dot{Q}}_{\mathrm{C}}<0$, ${\dot{Q}}_{\mathrm{H}}>0$ and ${\dot{W}}<0$ in a small time interval) as revealed in Langevin-dynamics models^48^. In order to inspect such fluctuation phenomena it is mandatory to evaluate quantities such as the power and performance of the nano machine along individual, short time intervals. As a first approach in this direction, we evaluate the fluctuations of the stochastic power from the MD simulations using the positional fluctuations of the center of mass of the nanoparticles. In particular, we evaluate the stochastic power exerted in a small time interval $[t,t+\mathrm{d}t]$ using the expression from stochastic thermodynamics^9^ associated with the model given by (1)

8 $$\begin{aligned} \dot{W} = \underbrace{\kappa _{\mathrm{C}} X_{\mathrm{H}} \circ {\dot{X}}_{\mathrm{C}}}_{=\displaystyle {\dot{W}}_{\mathrm{C}}} + \underbrace{\kappa _{\mathrm{H}} X_{\mathrm{C}}\circ {\dot{X}}_{\mathrm{H}}}_{=\displaystyle {\dot{W}}_{\mathrm{H}}}. \end{aligned}$$

Here, $\circ$ the Stratonovich product, whereas ${\dot{X}}_{\mathrm{C}} =[X_{\mathrm{C}} (t+\mathrm{d}t) - X_{\mathrm{C}} (t)]/\mathrm{d}t$ and ${\dot{X}}_{\mathrm{H}} =[X_{\mathrm{H}} (t+\mathrm{d}t) - X_{\mathrm{H}} (t)]/\mathrm{d}t$ are the time-averaged velocities estimated from the positions of the nanoparticles. We have also introduced ${\dot{W}}_{\mathrm{C}}$ and ${\dot{W}}_{\mathrm{H}}$ as the fluctuating power exerted on the cold (hot) nanoparticle in the interval $[t,t+\mathrm{d}t]$ respectively. Note that in (8) we take into account only the non-conservative forces exerted on each nanoparticle due to their nonreciprocal coupling.

**Figure 5:**
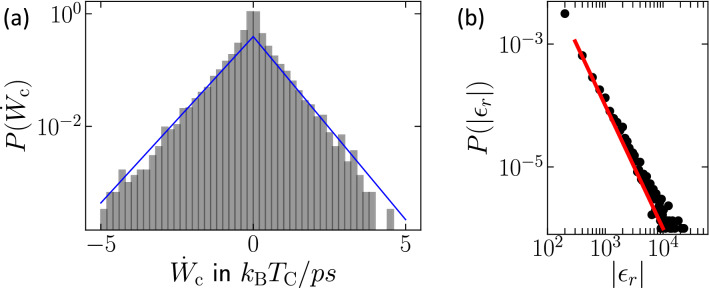
Fluctuations of stochastic power and efficiency. (**a**) Distribution of the power exerted on the cold nanoparticle: result from the MD simulations (bars) and analytical prediction given by (9) (solid blue line). Note that the lower panel in Fig. 2b displays the corresponding values of $W_{\mathrm{C}}$. (**b**) Distribution of the stochastic coefficient of performance defined by (10): empirical density obtained from the MD simulations (see Methods section) and theoretical prediction given by a power law with exponent $-2$ (red line). For the second panel we took $\kappa _{\mathrm{H}}/\kappa _{\mathrm{C}}=7.27$ and fixed the rest of the parameter values the same as in Fig. 3.

We now evaluate the stochastic power (8) from our MD simulations over time intervals of duration $\mathrm{d}t=1$ps. To this aim, we extract the empirical probability density for the stochastic power which reveals considerable fluctuations (gray bars in Fig. 5a). The distribution estimated from the MD simulations can be described with impressive accuracy using the closed-form expressions for the distribution of $\dot{W}_{\mathrm{C}}$ and $\dot{W}_{\mathrm{H}}$

9 $$\begin{aligned} P(\dot{W}_j)= \frac{1}{{\mathcal {Z}}_j} \,\exp \left[ \frac{\beta _j}{\kappa _j} {\dot{W}}_j -2\frac{\sqrt{\zeta _j \alpha _j}}{|\kappa _j|} |{\dot{W}}_j| \right] , \end{aligned}$$

which we derive analytically using the definition of the stochastic power (8) and assuming the effective stochastic model (1). In (9), $j\in \{\mathrm{C,H}\}$ is the particle label, ${\mathcal {Z}}_j$ is a normalization constant, and $\alpha _j, \beta _j, \zeta _j$ are functions of $\kappa _{\mathrm{C}},\kappa _{\mathrm{H}},T_{\mathrm{C}},T_{\mathrm{H}},\gamma ,m$ (see Methods section for their explicit expressions). The power distributions have exponential tails and are slightly asymmetric. In the refrigerator mode, $P(\dot{W}_{\mathrm{C}})$ leans towards positive values, consistent with the net negative work value, while $P(\dot{W}_{\mathrm{H}})$ leans towards positive work values. Furthermore, we find a good agreement between the MD simulation results and (9) throughout the parameter regime that we explore. The theoretical predictions yield a systematic overestimation of the variance which we attribute to the finite timestep $\mathrm{d}t$ in the MD simulations, while in the theoretical calculations we assume it to be infinitesimal (see Supplemental Material for the values of the variance of the power for different values of $\kappa _{\mathrm{H}}/\kappa _{\mathrm{C}}$).

The previous analyses showing that the work and heat in a small time interval are highly fluctuating motivates us to investigate the finite-time fluctuations of the coefficient of performance of the nano machine. To quantify how much the efficiency fluctuates for individual trajectories, we consider the *stochastic* coefficient of performance defined as^49^

10 $$\begin{aligned} \varepsilon _r =&\frac{|{\dot{Q}}_{\mathrm{C}}|}{{\dot{W}}}. \end{aligned}$$

Note that since in general $\langle |{\dot{Q}}_{\mathrm{C}}|\rangle /\langle \dot{W}\rangle \ne \langle |{\dot{Q}}_{\mathrm{C}}|/{\dot{W}}\rangle$, the ensemble average of $\varepsilon _r$ does not coincide with the average COP given by (7). Figure 5b displays the empirical distribution of the stochastic COP obtained from the MD simulations, which develops a fat tail with values that can exceed significantly the Carnot value $\varepsilon _{\mathrm{C}} =T_{\mathrm{C}}/ (T_{\mathrm{H}}-T_{\mathrm{C}})=5$. Such super-Carnot performance achieved in short time intervals have also been reported in experimental conditions and theoretical models of nanoscopic heat engines^50–53^ and nano refrigerators^48^; they result from rare events where the refrigerator works transiently as a heater reversing the work flux with respect to its average behaviour. Furthermore we find that the distribution of $\varepsilon _r$ extracted from the MD simulations follows in good approximation a power law

11 $$\begin{aligned} P(\varepsilon _r) \simeq |\varepsilon _r|^{-2}, \end{aligned}$$

See red line in Fig. 5b. The power-law behavior that we find reinforces the critical significance of thermal fluctuations for our nano machine system setup. Remarkably, we find that the power law (11) is in good agreement with the numerical results for all values of $\kappa _{\mathrm{H}}$ and $\kappa _{\mathrm{C}}$ that we explored, suggesting a universal scaling behavior as predicted by previous theoretical work within the realm of stochastic efficiency^50^.

#### Uncertainty relations

The results in previous sections revealed the instrumental role of stochastic thermodynamics to establish design principles for the parameter values of the nano machine to achieve prescribed values of the net power and efficiency. In the following, we investigate how one can use principles from stochastic thermodynamics—namely the so-called thermodynamic uncertainty relations^38,39,54^—to put fundamental constraints that regulate the trade-off between dissipation and precision of the nano machine.

**Figure 6:**
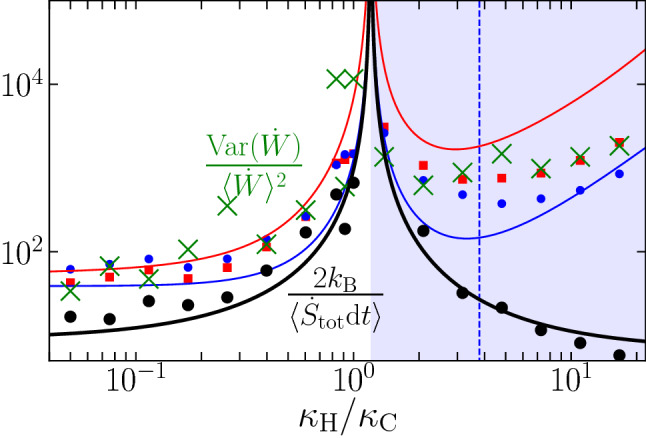
Thermodynamic uncertainty relation for power fluctuations. Relative uncertainty $\text {Var}({\dot{W}})/\langle {\dot{W}}\rangle ^2$ of the stochastic power exerted on the cold ${\dot{W}}_{\mathrm{C}}$ (blue filled circles) and the hot ${\dot{W}}_{\mathrm{H}}$ (red filled squares) nanoparticles, computed using in Eq. (8) the output of our MD simulations. The uncertainty of the total power on the two-nanoparticle setup ${\dot{W}}={\dot{W}}_{\mathrm{C}}+{\dot{W}}_{\mathrm{H}}$ is shown by green crosses. The blue and red lines are given by our theoretical predictions for the relative uncertainty of ${\dot{W}}_{\mathrm{C}}$ and ${\dot{W}}_{\mathrm{H}}$, respectively (see Supplemental for the full derivation). The relative uncertainty of the stochastic power lies above 2 divided by the average entropy production during $\mathrm{d}t$ [black symbols, MD simulations; black line, theoretical prediction given by (5)], in agreement with the thermodynamic uncertainty relation (12). The dashed vertical line depicts the theoretical value of $\kappa _{\mathrm{H}}/\kappa _{\mathrm{C}}=3.78843$ at which the heat extraction from the cold bath is maximal.

A suitable measure to quantify the strength of the power fluctuations is the *uncertainty* of the power, defined by the variance over the squared mean. High values of uncertainty indicate that the dynamics is essentially dominated by fluctuations. We have measured the uncertainty of the total power $\dot{W} = \dot{W}_{\mathrm{C}} + \dot{W}_{\mathrm{H}}$ in the MD simulations from individual trajectories, by making a statistics over the extracted values of the work. Figure 6 shows the results for the uncertainty of the total power (green symbols), as well as the power uncertainties of ${\dot{W}}_{\mathrm{C}}$ and ${\dot{W}}_{\mathrm{H}}$ separately (blue and the red symbols). Remarkably, the uncertainties reach extremely high values around the pseudo equilibrium point, and even seem to diverge at $\kappa _{\mathrm{H}} /\kappa _{\mathrm{C}} = T_{\mathrm{H}}/T_{\mathrm{C}}$. As we show below, this blow-up can be understood by making use of a recently-developed trade-off relation between the precision of thermodynamic currents and the rate of entropy production.

In the field of stochastic thermodynamics, recently a universally class of results—often called e thermodynamic uncertainty relation—governing Markovian nonequilibrium stationary states were derived, see e.g.^38,39,54^. Such laws connect the uncertainty of a current, quantified by its signal-to-noise ratio, with the total thermodynamic cost, measured by the steady-state rate of entropy production. In particular, the thermodynamic uncertainty relation in Ref.^55^ implies that the uncertainty of the finite-time power fluctuations of stationary Markovian processes is always bounded from below by $2k_{\mathrm{B}}$ over the mean total entropy production during $\mathrm{d}t$, i.e.,

12 $$\begin{aligned} \frac{\mathrm{Var}({{\dot{W}}})}{\langle \dot{W} \rangle ^2} \ge \frac{2 k_{\mathrm{B}}}{\langle \dot{S}_{\mathrm{tot}}\rangle \mathrm{d}t}. \end{aligned}$$

Equation (12) reveals that there exists a minimal thermodynamic cost associated with achieving a certain precision of the power exerted by the controller. Applying this law to the present case, we find from (5) that $\langle \dot{S}_{\mathrm{tot}} \rangle \rightarrow 0$ at $\kappa _{\mathrm{H}} /\kappa _{\mathrm{C}} \rightarrow T_{\mathrm{H}}/T_{\mathrm{C}}$, thus, the power uncertainty indeed diverges at the pseudo equilibrium point. We have complemented the MD simulation results in Fig. 6 with a black line showing the lower bound $2 k_{\mathrm{B}}/\langle \dot{S}_{\mathrm{tot}}\rangle \mathrm{d}t$ according to (12), which provides the first test of thermodynamic uncertainty relations in nonequilibrium MD setups.

From the simulation results shown in Fig. 6, we further detect a minimum in the total power uncertainty in the refrigerator regime. Interestingly, the position of this minimum (that is $\kappa _{\mathrm{H}}/\kappa _{\mathrm{C}} \approx 3.3$) roughly coincides with the $\kappa _{\mathrm{H}}/\kappa _{\mathrm{C}}$ value where the amount of extracted heat is maximal indicated by a vertical, dashed line in Fig. 6. For the chosen parameters, the theoretical value for the maximal heat extraction from the cold bath is $\kappa _{\mathrm{H}}/\kappa _{\mathrm{C}} \approx 3.79$, as obtained from differentiating (3). Thus, there is a regime where the refrigeration is maximal while at the same time, the power to sustain the refrigerator is as precise as possible.

## Discussion

We have shown with molecular dynamics simulations that it is possible to attain a net heat flow from a cold to a hot bath by connecting two nanoparticles immersed in fluid containers through nonreciprocal forces. Such nonreciprocal forces could be realized in the laboratory upon using, e.g., feedback traps^56^ which allow to exert in real time forces based on measurements of only the position of the center of mass of the nanoparticles. This represents an advantage with respect to traditional Maxwell-demon approaches where the measurement of position and velocities of the bath molecules is required to attain a heat flow from a cold to a hot thermal bath. Notably, our setup is also advantageous with respect to previous theoretical proposals that require the usage of athermal fluctuations together with nonlinear forces between the nanoparticles^57^.

In our simulations we have studied the case of copper nanoparticles immersed in argon, however we expect this effect to be generic for a class of systems whose dynamics can be described by linear underdamped Langevin equations. For such class of systems, we have revealed by using stochastic thermodynamics the necessary conditions and therefore the design principles that ensure a reverse heat flow (from cold to hot) and provided predictions on key thermodynamic properties such as the net heat transfer and the coefficient of performance. Our theoretical results reveal that the refrigeration effect generated by the nonreciprocal coupling is robust. It can be achieved in a broad parameter range as long as the dynamics is stable (see the Supplemental Material for further information about the stability conditions).

This work demonstrates fruits of the bridge between molecular dynamics with stochastic thermodynamics, namely the possibility to establish quantitative criteria to control the statistics of thermodynamic fluxes in nanoparticle-based thermal machines. We have developed analytical formulae that describe the average heat fluxes between the nanoparticles, the entropy production rate and the coefficient of performance. Moreover, we have tackled analytically the statistics of the power and the coefficient of performance of the nano machine over short time intervals within fluctuations play a prominent role. From the theoretical perspective, we expect that stochastic-thermodynamic approaches could shed light in the future on optimal design of nano refrigerator devices taking into account e.g. the effect of delay and/or memory in the exertion of nonreciprocal forces that may play a key role in realistic experimental scenarios.

In the current era when efficient energy conversion is of paramount importance, optimizing thermal transport in both classical and quantum systems has become a topic of intense theoretical and experimental interest for nanotechnology^58,59^. Furthermore, the possibility of realizing thermal conduits in various biological systems have been proposed to optimize thermal networks and information transfer^60,61^. Transient reverse heat flow in these contexts may confer biological machinery with enhanced functionality for energy harvesting. In this regard, it would be interesting to theoretically investigate whether our theoretical and atomistic setup can be used to generate heat flows in particles immersed in more complex environments including viscoelastic fluids, or even active (e.g. bacterial) baths.

## Methods

### MD simulations

We employed the LAMMPS for performing all out MD simulations^62^. The interatomic force between atoms was accounted by a Lennard–Jones (LJ) potential function,

13 $$\begin{aligned} \phi (r_{ij})=4\varepsilon [(\sigma /r_{ij} )^2- (\sigma /r_{ij} )^6 ], \end{aligned}$$

where $r_{ij}$ is the interatomic distance between atom *i* to atom *j*, $\varepsilon$ is the depth of the potential well, and $\sigma$ the distance at which the particle–particle potential energy vanishes. The LJ parameter values $\varepsilon$ and $\sigma$ of both argon–argon and copper–copper interactions are summarized in Table 1. The parameters have been chosen from earlier literature that compared the simulation outputs with experimental data or quantum calculations and were extensively utilized in many past studies^63–68^. The selected LJ parameters for argon reproduce the density of liquid argon in good agreement with experimental measurements and also the phononic properties of solid argon at low temperatures^63^. The LJ potential coefficients for copper correctly reproduce the crystalline state properties at various temperatures^64,65^.

**Table 1:** LJ interaction parameters.

	$\varepsilon (\mathrm{eV})$	$\sigma ($Å)
Ar–Ar	0.0104	3.405
Cu–Cu	0.4093	2.338

For the interatomic forces between argon and copper atoms, we employed the Lorentz–Berthelot mixing rules^69^, i.e.

14 $$\begin{aligned} \varepsilon _{12} = \sqrt{\varepsilon _1\, \varepsilon _2}, \sigma _{12}= (\sigma _2+ \sigma _1)/2. \end{aligned}$$

As shown in Fig. 1, our setup consists of two containers of cold and hot (defined by blue and red colours respectively) argon–copper mixture, each with volume $11 \mathrm{nm}^3$ and containing 25280 Ar fluid atoms with total mass $m _{\mathrm{Ar}}= 1.67\times 10^{-21} \mathrm{kg}$. The nanoparticles are made each of 186 Cu atoms with nanoparticle mass $m= 1.96\times 10^{-23} \mathrm{kg}$ and radius $r = 1.44 \mathrm{nm}$. To prevent interactions between the two containers and separate cold and hot baths, one-atom-thick Cu walls are placed between the containers, in the edges and in the middle of the simulation box along the *X* direction. Ar atoms of one container do not interact with the Ar atoms inside the other container. Periodic boundary condition were applied in the *Y* and *Z* directions while the *X* direction is constrained by the walls. The simulations were carried out for $15-30 \mathrm{ns}$. Within this time the temperature of fluids at both hot and cold baths were controlled at $T_{\mathrm{H}}=120\mathrm{K}$ and $T_{\mathrm{C}}=100\mathrm{K}$ using Nosé–Hoover thermostats (NVT) with a coupling time-constant of $1\mathrm{ps}$. We have also checked that the reversed heat flow setup is not sensitive to the choice of Nosé–Hoover as it is also reproduced using a Langevin-based thermostat as well (data not shown). A time step of $1 \mathrm{fs}$ was chosen for the MD simulations and the data was sampled every $1\mathrm{ps}$.

### Estimation of the effective friction coefficient

The noise terms as well as the friction forces appearing in the Langevin equations are not explicitly present in the MD simulations, but they emerge implicitly from the interactions between nanoparticle and surrounding bath particles. In our stochastic model, we assume Stokes law, in particular, instantaneous effective friction force that is linearly proportional on the instantaneous velocity. We obtain estimates for the values of the corresponding friction coefficients $\gamma _{\mathrm{C,H}}$ from MD simulations in the case of no coupling, $\kappa _{\mathrm{C,H}}=0$, via two distinct routes. First, we measure the velocity and positional autocorrelation functions in an equilibrium MD simulation, and fit the corresponding analytical expression taken from Ref.^70^:

15 $$\begin{aligned} \!\!\frac{\langle {X}_\mu (t){X}_\mu (t+\tau )\rangle }{\langle {X}_\mu ^2 (t)\rangle }\!\!= & {} \!\!\exp \left( -\frac{\gamma _\mu \tau }{2 m}\right) \left[ \cos ( \omega _\mu \tau ) + \frac{\gamma \sin (\omega _\mu \tau )}{2 m \omega _\mu } \right] \nonumber \\ \!\!\frac{\langle {{\dot{X}}}_\mu (t){{\dot{X}}}_\mu (t+\tau )\rangle }{\langle {{\dot{X}}}_\mu ^2 (t)\rangle }\!\!= & {} \!\! \exp \left( -\frac{\gamma _\mu \tau }{2 m}\right) \left[ \cos ( \omega _\mu \tau ) -\frac{\gamma \sin (\omega _\mu \tau )}{2 m \omega _\mu } \right] , \end{aligned}$$

with $\omega _\mu ^2 =(\kappa /m)-(\gamma _\mu /m)^2$, and we recall $\mu =\{{\mathrm{C,H}}\}$. Equation (15) reproduce our numerical estimates for both the position and velocity autocorrelation functions in equilibrium conditions (see the Supplemental for further details). Fitting (15) to our numerical results by setting *m*, $\kappa$ to their input values in the simulations, we extract the estimates $\gamma _{\mathrm{C}}\simeq \gamma _{\mathrm{H}} \sim 3.5 \times 10^{-12}$ kg/s for the effective friction coefficient. We use this estimate for $\gamma$ and set $\gamma _{\mathrm{C}} = \gamma _{\mathrm{H}}$. Second, we have performed a dragging experiment, pulling the nanoparticle through the bath and measure the resulting velocity (see the Supplemental Material for further details), which yields an estimate of $\gamma _{\mathrm{C}} \approx \gamma _{\mathrm{H}} \approx 3\times 10^{-12} \text {kg/s}$. Both routes yield consistent results. We use $\gamma =\gamma _{\mathrm{C}} = \gamma _{\mathrm{H}} = 3.5\times 10^{-12} \text {kg/s}$ throughout the paper.

### Definition of heat and work in stochastic thermodynamics

We address the thermodynamics of the nanoparticle system by applying the framework of stochastic thermodynamics^9,11^ to our stochastic model. For convenience, we first rewrite Eqs. (1) and (2) as a two-dimensional Langevin equation

16 $$\begin{aligned} m\ddot{\textbf{X}} = -\gamma \dot{\textbf{X}} -\nabla E+ \textbf{F}_{\mathrm{nr}} + \Xi \end{aligned}$$

where $\textbf{X}= ( X_{\mathrm{C}}, X_{\mathrm{H}} )^{{\textsf{T}}}$, $\Xi =(\xi _{\mathrm{C}},\xi _{\mathrm{H}})^{{\textsf{T}}}$, and $\nabla = (\partial _{X_{\mathrm{C}}},\partial _{X_{\mathrm{H}}})^{{\textsf{T}}}$, with ${{\textsf{T}}}$ denoting transposition. The term $\textbf{F}_{\mathrm{nr}}=(\kappa _{\mathrm{C}} X_{\mathrm{H}}, \kappa _{\mathrm{H}} X_{\mathrm{C}})^{{\textsf{T}}}$ is the nonreciprocal part of the total force acting on the system, which is non-conservative. On the other hand, the energy *E* of the nanoparticles

17 $$\begin{aligned} E = \frac{m}{2}({\dot{X}}_{\mathrm{C}}^2 + {\dot{X}}_{\mathrm{H}}^2) + \left( \frac{\kappa +\kappa _{\mathrm{C}}}{2}\right) X_{\mathrm{C}}^2 + \left( \frac{\kappa +\kappa _{\mathrm{H}}}{2}\right) X_{\mathrm{H}}^2 \end{aligned}$$

is a function of the instantaneous values of the particles’ positions and velocities, and thus a quantity that fluctuates during a simulation, i.e. a stochastic process. Following Sekimoto^9^, the energy change in a small time interval $[t,t+\mathrm{d}t]$ can be written as

18 $$\begin{aligned} \mathrm{d}E = \nabla E(\textbf{X}) \circ \mathrm{d}{} \textbf{X} + m\ddot{\textbf{X}}\circ \mathrm{d}{\textbf{X}}, \end{aligned}$$

where $\circ$ denotes the Stratonovich product. Here, $\mathrm{d}{} \textbf{X}= ( \mathrm{d}X_{\mathrm{C}} , \mathrm{d}X_{\mathrm{H}} )^T$ is the stochastic increment of the nanoparticles’ positions which follows from the Langevin dynamics (16). In $[t,t+\mathrm{d}t]$ the stochastic work done on the system is given by the non-conservative force times the displacement of the nanoparticles, which we can split as

19 $$\begin{aligned} \mathrm{d}W = \mathbf{F_{\mathrm{nr}}}\circ \mathrm{d}{\textbf{X}} = \underbrace{\kappa _{\mathrm{C}} X_{\mathrm{H}} \circ \mathrm{d}X_{\mathrm{C}}}_{=\displaystyle \mathrm{d}W_{\mathrm{C}}} + \underbrace{\kappa _{\mathrm{H}} X_{\mathrm{C}}\circ \mathrm{d}X_{\mathrm{H}}}_{=\displaystyle \mathrm{d}W_{\mathrm{H}}}, \end{aligned}$$

where $\mathrm d W_{\mathrm{C}}$ ($\mathrm d W_{\mathrm{H}}$) is the work done on the cold (hot) nanoparticle. Dividing the stochastic work $\mathrm{d}W$ applied to the particle along a trajectory of length $\mathrm{d}t$ yields the power ${\dot{W}}= \mathrm{d}{W}/\mathrm{d}t$.

Similarly, the stochastic heat dissipated in the same time interval

20 $$\begin{aligned} \mathrm{d} Q = \mathrm{d}E - \mathrm{d}W = \underbrace{(\gamma _\mu {\dot{X}}_{\mathrm{C}} - \xi _{\mathrm{C}} )\circ \mathrm{d}{X}_{\mathrm{C}}}_{=\displaystyle \mathrm{d}Q_{\mathrm{C}}} + \underbrace{(\gamma _\mu {\dot{X}}_{\mathrm{H}} - \xi _{\mathrm{H}} )\circ \mathrm{d}{X}_{\mathrm{H}}}_{=\displaystyle \mathrm{d}Q_{\mathrm{H}}}, \end{aligned}$$

which ensures that the first law is satisfied for every single trajectory traced by the system. Note that we use the thermodynamic sign convention: $W>0$ ($W<0$) when work is exerted (extracted) from the system and $Q>0$ ($Q<0$) when heat is dissipated from (absorbed by) the system to (from) its environment. Dividing the stochastic heat dissipated along a trajectory of length by the trajectory length $\mathrm{d} t$ yields the stochastic heat dissipation rate ${\dot{Q}}= \mathrm{{d}} {Q}/\mathrm{d}t$.

### Power fluctuations

The power fluctuations of each nanoparticle predicted by our stochastic model are given in Eq. (9) as closed-form expressions for $P(\dot{W}_j)$ with $j\in \{ \text {C,H} \}$. The distributions $P(\dot{W}_j)$ explicitly depend on $\alpha _j,\beta _j,\zeta _j$, which are in turn functions of the covariances of the positions and velocities of the *j* nanoparticle and the $l\ne j$ nanoparticle. Specifically,

21 $$\begin{aligned} \alpha _j&= \frac{1}{2 \langle X_l^2 \rangle (1-\psi _j^2)}, ~~~~ \beta _j = \frac{\psi _j}{\sqrt{\langle X_l^2 \rangle \langle \dot{X}_j^2 \rangle }(1-\psi ^2_j)},~~~~\zeta _j = \frac{1}{2 \langle \dot{X}_j^2 \rangle (1-\psi _j^2)}, ~~~~ \psi _j=\frac{\langle X_l {\dot{X}}_j \rangle }{\sqrt{\langle X_l^2 \rangle \langle \dot{X}_j^2 \rangle }}, \end{aligned}$$

with

22 $$\begin{aligned} \langle {X}_l^2 \rangle&= k_{\mathrm{B}} \frac{ m T_l [\kappa _j^2 (\kappa _j+\kappa _l) + \kappa (\kappa _j^2 + \kappa _l^2)] + 2\gamma ^2 [\kappa _l^2 T_j + T_l(\kappa +\kappa _j)(2\kappa +\kappa _j) +\kappa \kappa _l T_l] + m T_j \kappa _l^2 [2 \kappa + \kappa _j + \kappa _l] }{\kappa (\kappa + \kappa _j + \kappa _l)[m \left[ (\kappa _j + \kappa _l)^2 +2 (2\kappa + \kappa _j + \kappa _l)\right] ]}\, , \end{aligned}$$

23 $$\begin{aligned} \langle {\dot{X}}_j^2 \rangle&= k_{\mathrm{B}}\frac{ \kappa _j (\kappa _j T_l-\kappa _l T_j)}{(m/2) (\kappa _j + \kappa _l)^2+ \gamma ^2 (2\kappa +\kappa _j+\kappa _l)}+\frac{k_{\mathrm{B}} T_j}{m}\,, \end{aligned}$$

and

24 $$\begin{aligned} \langle {X}_l{{\dot{X}}}_j \rangle = k_{\mathrm{B}} \frac{ 2\gamma (\kappa _j T_l - \kappa _l T_j )}{m (\kappa _j+\kappa _l)^2+ 2(2\kappa + \kappa _j \kappa _l) \gamma ^2}\,. \end{aligned}$$

From the power distributions $P(\dot{W}_j)$, we can further deduce analytical expressions for the variance of the power:

25 $$\begin{aligned} \text {Var}(\dot{W}_j)&= \frac{1}{{\mathcal {Z}}_j}\!\left\{ \frac{2\kappa _j^3}{(2\sqrt{\alpha _j \zeta _j}+\beta _j)^3} + \frac{2\kappa _j^3}{(2\sqrt{\alpha _j \zeta _j}-\beta _j)^3} - \left[ \frac{\kappa _j^2}{(2\sqrt{\alpha _j \zeta _j}+\beta _j)^2} + \frac{2\kappa _j^2}{(2\sqrt{\alpha _j \zeta _j}-\beta _j)^2}\right] ^2 \right\} . \end{aligned}$$

See the Supplemental Material for further details and the explicit mathematical derivations.

## Supplementary Information

Supplementary Information.

## Data Availability

The datasets used and analysed during the current study available from the corresponding author on reasonable request.

## References

[1] Bustamante, C., Liphardt, J. & Ritort, F. The nonequilibrium thermodynamics of small systems. http://arxiv.org/abs/cond-mat/0511629 (2005).

[2] Ashkin A, Dziedzic JM, Bjorkholm JE, Chu S (1986). Observation of a single-beam gradient force optical trap for dielectric particles. Opt. Lett..

[3] Haroche S, Brune M, Raimond J (1991). Trapping atoms by the vacuum field in a cavity. EPL Europhys. Lett..

[4] Bechhoefer J (2005). Feedback for physicists: A tutorial essay on control. Rev. Mod. Phys..

[5] Toyabe S, Sagawa T, Ueda M, Muneyuki E, Sano M (2010). Experimental demonstration of information-to-energy conversion and validation of the generalized jarzynski equality. Nat. Phys..

[6] Campisi M, Pekola J, Fazio R (2017). Feedback-controlled heat transport in quantum devices: Theory and solid-state experimental proposal. New J. Phys..

[7] Ciliberto S (2020). Autonomous out-of-equilibrium Maxwell‘s demon for controlling the energy fluxes produced by thermal fluctuations. Phys. Rev. E.

[8] Parrondo JM, Espanol P (1996). Criticism of Feynman’s analysis of the ratchet as an engine. Am. J. Phys..

[9] Sekimoto K (2010). Stochastic Energetics.

[10] Jarzynski C (2011). Equalities and inequalities: Irreversibility and the second law of thermodynamics at the nanoscale. Annu. Rev. Condens. Matter Phys..

[11] Seifert U (2012). Stochastic thermodynamics, fluctuation theorems and molecular machines. Rep. Prog. Phys..

[12] Van den Broeck C, Esposito M (2015). Ensemble and trajectory thermodynamics: A brief introduction. Physica A.

[13] Peliti L, Pigolotti S (2021). Stochastic Thermodynamics: An Introduction.

[14] Gieseler J, Deutsch B, Quidant R, Novotny L (2012). Subkelvin parametric feedback cooling of a laser-trapped nanoparticle. Phys. Rev. Lett..

[15] Gieseler J, Quidant R, Dellago C, Novotny L (2014). Dynamic relaxation of a levitated nanoparticle from a non-equilibrium steady state. Nat. Nanotechnol..

[16] Rajabpour A (2019). Thermal transport at a nanoparticle-water interface: A molecular dynamics and continuum modeling study. J. Chem. Phys..

[17] Todd BD, Daivis PJ (2017). Nonequilibrium molecular dynamics: Theory, algorithms and applications.

[18] Roodbari M, Abbasi M, Arabha S, Gharedaghi A, Rajabpour A (2022). Interfacial thermal conductance between tio2 nanoparticle and water: A molecular dynamics study. J. Mol. Liq..

[19] Müller-Plathe F (1997). A simple nonequilibrium molecular dynamics method for calculating the thermal conductivity. J. Chem. Phys..

[20] Allen MP, Tildesley DJ (2017). Computer Simulation of Liquids.

[21] Fruchart M, Hanai R, Littlewood PB, Vitelli V (2021). Non-reciprocal phase transitions. Nature.

[22] Saha S, Agudo-Canalejo J, Golestanian R (2020). Scalar active mixtures: The nonreciprocal Cahn–Hilliard model. Phys. Rev. X.

[23] You Z, Baskaran A, Marchetti MC (2020). Nonreciprocity as a generic route to traveling states. Proc. Natl. Acad. Sci..

[24] Lavergne FA, Wendehenne H, Bäuerle T, Bechinger C (2019). Group formation and cohesion of active particles with visual perception-dependent motility. Science.

[25] Loos, S. A. M., Klapp, S. H. L. & Martynec, T. Long-range order and directional defect propagation in the nonreciprocal xy model with vision cone interactions. http://arxiv.org/abs/2206.10519 (2022). 10.1103/PhysRevLett.130.198301 37243650

[26] Braverman L, Scheibner C, VanSaders B, Vitelli V (2021). Topological defects in solids with odd elasticity. Phys. Rev. Lett..

[27] Poncet A, Bartolo D (2022). When soft crystals defy newton’s third law: Nonreciprocal mechanics and dislocation motility. Phys. Rev. Lett..

[28] Loos SAM, Klapp SHL (2020). Irreversibility, heat and information flows induced by non-reciprocal interactions. N. J. Phys..

[29] Rieser J (2022). Tunable light-induced dipole-dipole interaction between optically levitated nanoparticles. Science.

[30] Maxwell JC (1986). Maxwell on Molecules and Gases.

[31] Mandal D, Jarzynski C (2012). Work and information processing in a solvable model of Maxwell’s demon. PNAS.

[32] Mandal D, Quan HT, Jarzynski C (2013). Maxwell’s refrigerator an exactly solvable model. Phys. Rev. Lett..

[33] Kanazawa K, Sano TG, Sagawa T, Hayakawa H (2015). Minimal model of stochastic athermal systems: Origin of non-gaussian noise. Phys. Rev. Lett..

[34] Proesmans K, Dreher Y, Gavrilov M, Bechhoefer J, Van den Broeck C (2016). Brownian duet: A novel tale of thermodynamic efficiency. Phys. Rev. X.

[35] Bérut A, Imparato A, Petrosyan A, Ciliberto S (2016). Stationary and transient fluctuation theorems for effective heat fluxes between hydrodynamically coupled particles in optical traps. Phys. Rev. Lett..

[36] Ricci F (2017). Optically levitated nanoparticle as a model system for stochastic bistable dynamics. Nat. Commun..

[37] Midtvedt B (2021). Fast and accurate nanoparticle characterization using deep-learning-enhanced off-axis holography. ACS Nano.

[38] Barato AC, Seifert U (2015). Thermodynamic uncertainty relation for biomolecular processes. Phys. Rev. Lett..

[39] Horowitz JM, Gingrich TR (2020). Thermodynamic uncertainty relations constrain non-equilibrium fluctuations. Nat. Phys..

[40] Dellago C, Hummer G (2014). Computing equilibrium free energies using non-equilibrium molecular dynamics. Entropy.

[41] Park S, Khalili-Araghi F, Tajkhorshid E, Schulten K (2003). Free energy calculation from steered molecular dynamics simulations using Jarzynski’s equality. J. Chem. Phys..

[42] Khadka U, Holubec V, Yang H, Cichos F (2018). Active particles bound by information flows. Nat. Commun..

[43] Stewart WE, Lightfoot EN, Bird RB (1962). Transport Phenomena.

[44] Kwon C, Ao P, Thouless DJ (2005). Structure of stochastic dynamics near fixed points. Proc. Natl. Acad. Sci..

[45] Bae Y, Lee S, Kim J, Jeong H (2021). Inertial effects on the Brownian gyrator. Phys. Rev. E.

[46] Kwon C, Noh JD, Park H (2011). Nonequilibrium fluctuations for linear diffusion dynamics. Phys. Rev. E.

[47] Borgnakke C, Sonntag RE (2022). Fundamentals of Thermodynamics.

[48] Rana S, Pal P, Saha A, Jayannavar A (2016). Anomalous Brownian refrigerator. Physica A.

[49] Joseph T, Kiran V (2021). Efficiency estimation for an equilibrium version of the Maxwell refrigerator. Phys. Rev. E.

[50] Polettini M, Verley G, Esposito M (2015). Efficiency statistics at all times: Carnot limit at finite power. Phys. Rev. Lett..

[51] Verley G, Esposito M, Willaert T, Van den Broeck C (2014). The unlikely Crnot efficiency. Nat. Commun..

[52] Martínez IA, Roldán É, Dinis L, Rica RA (2017). Colloidal heat engines: A review. Soft Matter.

[53] Park J-M, Chun H-M, Noh JD (2016). Efficiency at maximum power and efficiency fluctuations in a linear Brownian heat-engine model. Phys. Rev. E.

[54] Hasegawa Y, Van Vu T (2019). Uncertainty relations in stochastic processes: An information inequality approach. Phys. Rev. E.

[55] Pietzonka P, Ritort F, Seifert U (2017). Finite-time generalization of the thermodynamic uncertainty relation. Phys. Rev. E.

[56] Jun Y, Bechhoefer J (2012). Virtual potentials for feedback traps. Phys. Rev. E.

[57] Kanazawa K, Sagawa T, Hayakawa H (2013). Heat conduction induced by non-Gaussian athermal fluctuations. Phys. Rev. E.

[58] Ercole L, Marcolongo A, Baroni S (2017). Accurate thermal conductivities from optimally short molecular dynamics simulations. Sci. Rep..

[59] Isaeva L, Barbalinardo G, Donadio D, Baroni S (2019). Modeling heat transport in crystals and glasses from a unified lattice-dynamical approach. Nat. Commun..

[60] Thompson EJ, Paul A, Iavarone AT, Klinman JP (2021). Identification of thermal conduits that link the protein–water interface to the active site loop and catalytic base in enolase. J. Am. Chem. Soc..

[61] Gao S, Klinman JP (2022). Functional roles of enzyme dynamics in accelerating active site chemistry: Emerging techniques and changing concepts. Curr. Opin. Struct. Biol..

[62] Plimpton S (1995). Fast parallel algorithms for short-range molecular dynamics. J. Comput. Phys..

[63] Stark J (1966). Fundamentals of Statistical Thermodynamics.

[64] Halicioglu T, Pound G (1975). Calculation of potential energy parameters form crystalline state properties. Phys. Status Solidi A.

[65] Yu J, Amar JG (2002). Effects of short-range attraction in metal epitaxial growth. Phys. Rev. Lett..

[66] Rajabpour A, Akizi FY, Heyhat MM, Gordiz K (2013). Molecular dynamics simulation of the specific heat capacity of water-cu nanofluids. Int. Nano Lett..

[67] Sanders DE, DePristo AE (1992). Predicted diffusion rates on fcc (001) metal surfaces for adsorbate/substrate combinations of Ni, Cu, Rh, Pd, Ag, Pt, Au. Surf. Sci..

[68] Sarkar S, Selvam RP (2007). Molecular dynamics simulation of effective thermal conductivity and study of enhanced thermal transport mechanism in nanofluids. J. Appl. Phys..

[69] Delhommelle J, Millié P (2001). Inadequacy of the Lorentz–Berthelot combining rules for accurate predictions of equilibrium properties by molecular simulation. Mol. Phys..

[70] Wang MC, Uhlenbeck GE (1945). On the theory of the Brownian motion II. Rev. Mod. Phys..

