# Supplementary material for: Nonreciprocal forces enable cold-to-hot heat transfer between nanoparticles
Source: Sci Rep. 2023 Mar 18;13:4517. doi: 10.1038/s41598-023-31583-y (PMC10024720; doi:10.1038/s41598-023-31583-y)
Supplement: Supplementary file 1 — Supplementary Information. [file 41598_2023_31583_MOESM1_ESM.pdf]

Supplemental Material to: Nonreciprocal forces enable cold-to-hot heat transfer between nanoparticles

Sarah A.M. Loos,[1,2] Saeed Arabha,[3,4] Ali Rajabpour,[4,5] Ali Hassanali,[2] and Édgar Roldán[2]

[1]*DAMTP, University of Cambridge, Wilberforce Road, Cambridge CB3 0WA, United Kingdom*
Electronic Address: sl2127@cam.ac.uk
[2]*ICTP – International Centre for Theoretical Physics, Strada Costiera, 11, 34151 Trieste, Italy*
[3]*Department of Mechanical Engineering, Lassonde School of Engineering, York University, Toronto, Canada*
[4]*Advanced Simulation and Computing Laboratory (ASCL),*
Imam Khomeini International University, Qazvin, Iran
[5]*School of Nano Science, Institute for Research in Fundamental Sciences (IPM), Tehran, Iran*

(Dated: February 27, 2023)

I. ESTIMATION OF FRICTION COEFFICIENTS

A. Dragging experiment in MD simulations

To estimate the friction coefficient, a dragging MD simulation is performed assuming the Stokes' law for the friction force ($F_{\rm friction} = -\gamma v$). In this regard, a constant force along the x axis of magnitude $F = 0.3$nN is applied to a spherical copper nanoparticle with radius $r = 1.44$nm immersed in an Ar fluid bath with fixed temperature 100K (first simulation) and 120K (second simulation) and periodic boundary conditions. As shown in Supplementary Fig. S1, the velocity of the Cu nanoparticle reached a steady value (terminal velocity) after a transient time of ~ 0.3ns and remained constant for the rest of the simulation. Using the Stokes' law, the friction coefficient is estimated from the ratio between the magnitude of the external force and the terminal velocity $\gamma = 2.8 \times 10^{-12}$kg/s obtained as the mean of the simulations done at the two temperature values. The simulation is repeated with an external force ten times larger ($F = 3$nN), yielding a very similar estimate of the friction coefficient $\gamma = 3.1 \times 10^{-12}$kg/s.

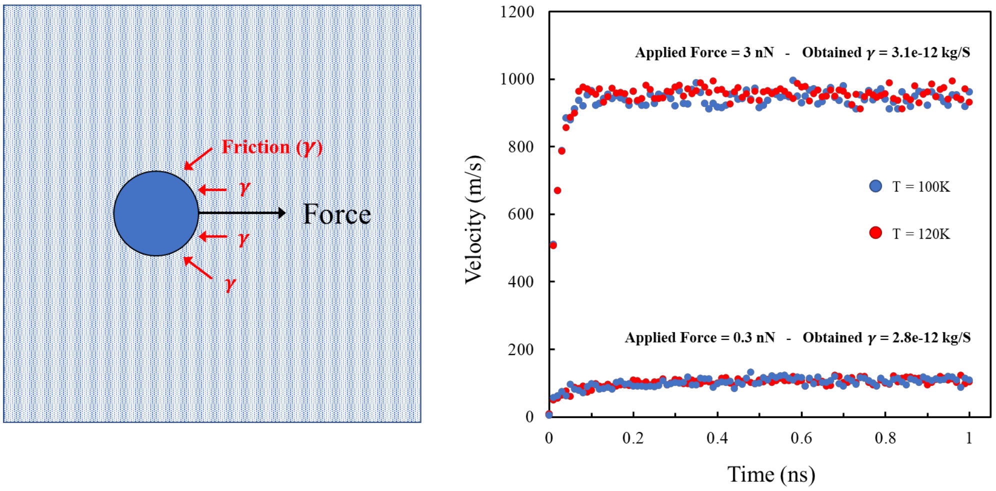

Supplementary Fig. S1: Left: Sketch of the dragging MD simulation used to estimate the friction coefficient (see text for details). Right: instantaneous velocity of the center of mass of the nanoparticle Assuming Stokes' law for the friction force $F_{\rm friction} = -\gamma v$, an estimate for the friction coefficient of $\gamma \sim 3 \times 10^{-12}$kg/s.

B. Equilibrium autocorrelation functions: MD simulations and Langevin theory

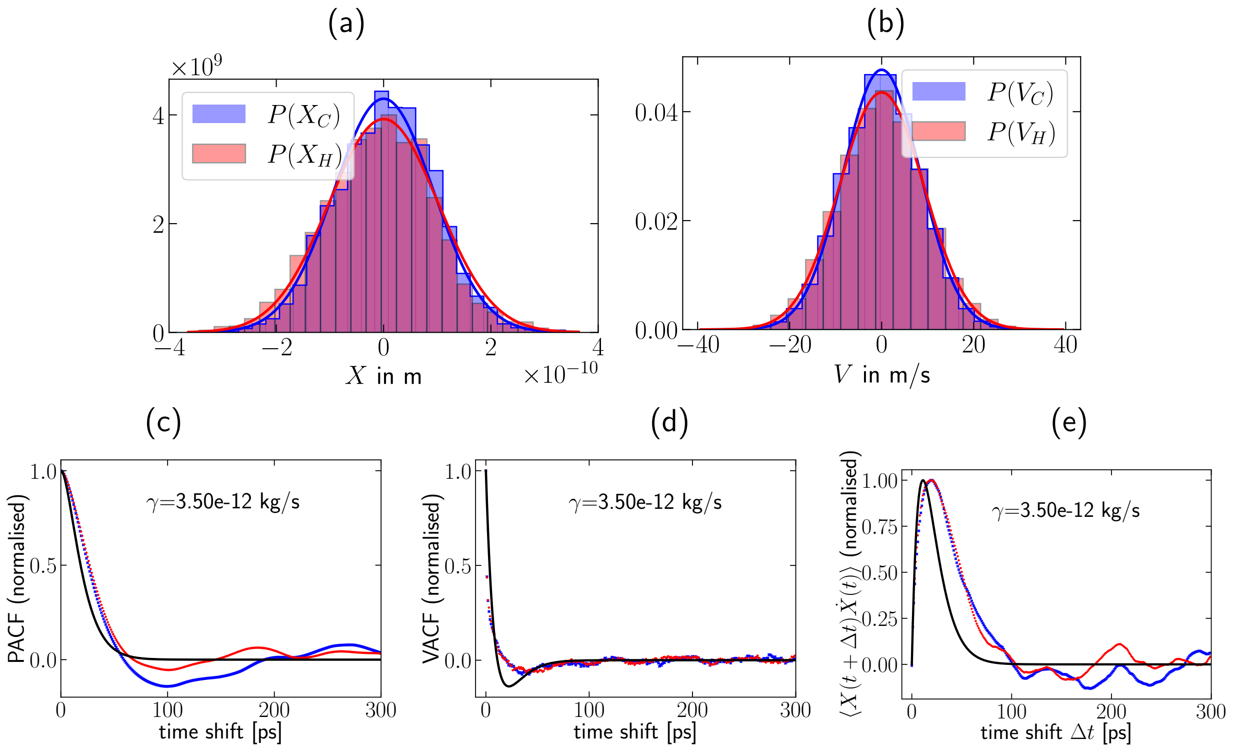

Supplementary Fig. S2: Fluctuations and correlations of position and velocity in the case of thermal equilibrium. (a,b) Positional and velocity fluctuations of nanoparticles in the uncoupled equilibrium case ($\kappa_{\rm H} = \kappa_{\rm C} = 0$). The bars are obtained from MD simulations and the lines show the theoretical predictions, i.e., zero-mean Gaussian functions with variance $k_{\rm B}T/a$, $k_{\rm B}T/m$, respectively, see Eq. (6). (c-e) Position autocorrelation function (PACF, c) and velocity autocorrelation function (VACF, d) and position-velocity cross correlation function (e) obtained from equilibrium MD simulations (with $\kappa_{\rm C} = \kappa_{\rm H} = 0$) extracted from the center-of-mass motion of the hot (red line) and cold nanoparticle (blue line). The black lines in (c-e) are fits to the analytical expressions for the underdamped Langevin model: Eq. (1) (c), Eq. (2) (d) and Eq. (3) (e) to the theoretical curves (3). The insets show the value of the effective friction extracted from the fits with only one free parameter, all of them yielding the estimate $\gamma \sim 3.5 \times 10^{-12}$kg/s.

Next, we describe how we extracted the effective friction coefficients from fits of the autocorrelation functions obtained from the MD simulations to analytical expressions derived below for our underdamped Langevin model. In thermal equilibrium, the normalised autocorrelation functions of the particle position and velocity are known. For $\kappa_{\rm C} = \kappa_{\rm H} = 0$, they read [1]

$$\mathrm{PACF}(\tau) = \exp\left(-\frac{\gamma\tau}{2m}\right)\left[\cos(\omega_1\tau) + \frac{\gamma}{2m\omega_1}\sin(\omega_1\tau)\right], \tag{1}$$

and

$$\mathrm{VACF}(\tau) = \exp\left(-\frac{\gamma\tau}{2m}\right)\left[\cos(\omega_1\tau) - \frac{\gamma}{2m\omega_1}\sin(\omega_1\tau)\right], \tag{2}$$

with $\omega_1 = \sqrt{\frac{\kappa}{m} - \frac{\gamma^2}{m^2}}$. Further, the position–velocity cross correlation is given by

$$\langle X(t)\dot{X}(t+\tau)\rangle = \exp\left(-\frac{\gamma\tau}{2m}\right)\left[\frac{\gamma\sin(\omega_1\tau)}{2m\omega_1}\right]. \tag{3}$$

Supplementary Fig. S2 displays MD simulation results from equilibrium simulations together with the fits to the theoretical predictions given by Eqs.(1-3).

II. ANALYTICAL CALCULATION OF HEAT AND ENTROPY PRODUCTION RATES

A. Heat flow rates

Here we calculate the ensemble-averaged heat flow rates $\langle \dot{Q}_j \rangle$, with $j \in \{\mathrm{C}, \mathrm{H}\}$. To this end, we start from Sekimoto's definition [2] of the stochastic heat dissipated to the j bath in $[t, t+\mathrm{d}t]$, i.e. $\mathrm{d}Q_j = (\gamma \dot{X}_j - \xi_j) \circ \mathrm{d}X_j$, and consider the steady-state average

$$\langle \dot{Q}_j \rangle = \left\langle \frac{\mathrm{d}Q_j}{\mathrm{d}t} \right\rangle = \gamma \langle \dot{X}_j^2 \rangle - \left\langle \xi_j \dot{X}_j \right\rangle . \tag{4}$$

Inserting $\langle \xi_j \dot{X}_j \rangle = \gamma k_\mathrm{B} T_j / m$, which follows from the Langevin equation [3], one finds

$$\langle \dot{Q}_j \rangle = \gamma \left[\langle \dot{X}_j^2 \rangle - \frac{k_\mathrm{B} T_j}{m} \right] . \tag{5}$$

Thus, the heat flow between each nanoparticle and its bath is directly given by the variance of its velocity fluctuations. We note that this formula can also be recast into the form $\langle \dot{Q}_j \rangle = \frac{k_\mathrm{B}\gamma}{m} \left[T_j^\mathrm{eff} - T_j \right]$, i.e., the heat flow is proportional to the difference between "effective temperature" $T_j^\mathrm{eff} = m \langle \dot{X}_j^2 \rangle / k_\mathrm{B}$ and actual temperature T_j.

The stationary joint probability density of the position and velocity of the two particles described by the linear Langevin equation [given in Eq (1) in the Main Text] is given by the four-variate normal distribution

$$\rho_4(x_\mathrm{C}, x_\mathrm{H}, v_\mathrm{C}, v_\mathrm{H}) = \frac{\exp\left[-\frac{1}{2} (\mathbf{r} - \langle \mathbf{r} \rangle)^\mathsf{T} \mathbf{C}^{-1} (\mathbf{r} - \langle \mathbf{r} \rangle) \right]}{\sqrt{(2\pi)^4 \det \mathbf{C}}} , \tag{6}$$

where $\mathbf{r} = (x_\mathrm{C}, x_\mathrm{H}, v_\mathrm{C}, v_\mathrm{H})^\mathsf{T}$ is a column vector with T denoting matrix transposition, $\langle \mathbf{r} \rangle$ its stationary average, and $\mathbf{C}$ the correlation matrix which is given by

$$\mathbf{C} = \begin{pmatrix} \langle X_\mathrm{C}^2 \rangle & \langle X_\mathrm{C} X_\mathrm{H} \rangle & \langle X_\mathrm{C} \dot{X}_\mathrm{C} \rangle & \langle X_\mathrm{C} \dot{X}_\mathrm{H} \rangle \\ \langle X_\mathrm{C} X_\mathrm{H} \rangle & \langle X_\mathrm{H}^2 \rangle & \langle X_\mathrm{H} \dot{X}_\mathrm{C} \rangle & \langle X_\mathrm{H} \dot{X}_\mathrm{H} \rangle \\ \langle \dot{X}_\mathrm{C} X_\mathrm{C} \rangle & \langle \dot{X}_\mathrm{C} X_\mathrm{H} \rangle & \langle \dot{X}_\mathrm{C}^2 \rangle & \langle \dot{X}_\mathrm{C} \dot{X}_\mathrm{H} \rangle \\ \langle \dot{X}_\mathrm{H} X_\mathrm{C} \rangle & \langle \dot{X}_\mathrm{H} X_\mathrm{H} \rangle & \langle \dot{X}_\mathrm{C} \dot{X}_\mathrm{H} \rangle & \langle \dot{X}_\mathrm{H}^2 \rangle \end{pmatrix} . \tag{7}$$

To calculate the variance of the velocity fluctuations, we develop further the formalism introduced in Refs. [4–6] to calculate correlation matrices. To this end, we first recast the Langevin equations into the matrix equations

$$\dot{\mathbf{r}} = -\mathbf{B}\,\mathbf{r} + \mathbf{\Xi} = -\begin{pmatrix} \mathbf{0_2} & -\mathbf{I_2} \\ -\mathbf{A}/m & \mathbf{\Gamma}/m \end{pmatrix} \mathbf{r} + \mathbf{\Xi}, \quad \mathbf{\Gamma} = \begin{pmatrix} \gamma & 0 \\ 0 & \gamma \end{pmatrix}, \quad \mathbf{A} = \begin{pmatrix} a_0 & k_0 \\ k_1 & a_1 \end{pmatrix}, \tag{8}$$

with the state vector $\mathbf{r} = (X_\mathrm{C}, X_\mathrm{H}, \dot{X}_\mathrm{C}, \dot{X}_\mathrm{H})^\mathsf{T}$ and the noise vector $\mathbf{\Xi} = (0, 0, \xi_\mathrm{C}, \xi_\mathrm{H})^\mathsf{T}$. The 4×4 matrix $\mathbf{B}$ contains the 2×2 zero and identity matrices, $\mathbf{0_2}$ and $\mathbf{I_2}$, the friction (diagonal) matrix $\mathbf{\Gamma}$ and the coupling matrix $\mathbf{A}$. For the sake of generality, we will here consider the most general case of coupling here (four independent coupling entries of the coupling matrix), and below specialize our results to $\mathbf{A} = \begin{pmatrix} -(\kappa + \kappa_\mathrm{C}) & \kappa_\mathrm{C} \\ \kappa_\mathrm{H} & -(\kappa + \kappa_\mathrm{H}) \end{pmatrix}$. Further, we define the diffusion matrices $\mathbf{D}_2 = \frac{k_\mathrm{B}\gamma}{m^2} \begin{pmatrix} T_\mathrm{C} & 0 \\ 0 & T_\mathrm{H} \end{pmatrix}$ and $\mathbf{D}_4 = \begin{pmatrix} \mathbf{0_2} & \mathbf{0_2} \\ \mathbf{0_2} & \mathbf{D}_2 \end{pmatrix}$. From these ingredients, we can determine the correlation matrix $\mathbf{C}$ from the following expressions:

$$\mathbf{C} = \mathbf{B}^{-1} (\mathbf{D}_4 + \mathbf{Q}) \tag{9}$$

where $\mathbf{Q}$ is an anti-symmetric 4×4 matrix that is uniquely determined by

$$\mathbf{B}\mathbf{Q} + \mathbf{Q}\mathbf{B}^\mathsf{T} = \mathbf{B}\mathbf{D}_4 - \mathbf{D}_4 \mathbf{B}^\mathsf{T} . \tag{10}$$

From this expression, and the fact that in stationary state $\langle \dot{X}_\mathrm{C} X_\mathrm{C} \rangle = \langle \dot{X}_\mathrm{H} X_\mathrm{H} \rangle = 0$, we obtain

$$\mathbf{C} = \begin{pmatrix} \langle X_\mathrm{C}^2 \rangle & \langle X_\mathrm{C} X_\mathrm{H} \rangle & 0 & \langle X_\mathrm{C} \dot{X}_\mathrm{H} \rangle \\ \langle X_\mathrm{C} X_\mathrm{H} \rangle & \langle X_\mathrm{H}^2 \rangle & \langle X_\mathrm{H} \dot{X}_\mathrm{C} \rangle & 0 \\ 0 & \langle \dot{X}_\mathrm{C} X_\mathrm{H} \rangle & \langle \dot{X}_\mathrm{C}^2 \rangle & \langle \dot{X}_\mathrm{C} \dot{X}_\mathrm{H} \rangle \\ \langle \dot{X}_\mathrm{H} X_\mathrm{C} \rangle & 0 & \langle \dot{X}_\mathrm{C} \dot{X}_\mathrm{H} \rangle & \langle \dot{X}_\mathrm{H}^2 \rangle \end{pmatrix} , \tag{11a}$$

with nonzero elements given by

$$\langle \dot{X}_{\rm C}^2\rangle = k_{\rm B}\frac{2k_0(k_0T_{\rm H}-k_1T_{\rm C})}{m\,[(a_0-a_1)^2+4k_0k_1]-2\gamma^2(a_0+a_1)}+\frac{k_{\rm B}T_{\rm C}}{m}, \tag{11b}$$

$$\langle \dot{X}_{\rm H}^2\rangle = k_{\rm B}\frac{2k_1(k_1T_{\rm C}-k_0T_{\rm H})}{m\,[(a_0-a_1)^2+4k_0k_1]-2\gamma^2(a_0+a_1)}+\frac{k_{\rm B}T_{\rm H}}{m}, \tag{11c}$$

$$\langle \dot{X}_{\rm C}X_{\rm H}\rangle = \langle \dot{X}_{\rm H}X_{\rm C}\rangle = k_{\rm B}\frac{(a_0-a_1)(k_1T_{\rm C}-k_0T_{\rm H})}{m\,[(a_0-a_1)^2+4k_0k_1]-2\gamma^2(a_0+a_1)}, \tag{11d}$$

$$\langle X_{\rm C}^2\rangle = k_{\rm B}\frac{mT_{\rm C}[-(a_0-a_1)^2a_1+(a_0-3a_1)k_0k_1]-k_0^2mT_{\rm H}(a_0+a_1)+2\gamma^2[a_1(a_0+a_1)T_{\rm C}-k_0k_1T_{\rm C}+k_0^2T_{\rm H}]}{(a_0a_1-k_0k_1)[m\,[(a_0-a_1)^2+4k_0k_1]-2\gamma^2(a_0+a_1)]}, \tag{11e}$$

$$\langle X_{\rm C}X_{\rm H}\rangle = k_{\rm B}\frac{mT_{\rm C}k_1[-a_0a_1+a_1^2+2k_0k_1]+k_0mT_{\rm H}(a_0^2-a_0a_1+2k_0k_1)+2\gamma^2(a_1k_1T_{\rm C}-a_0k_0T_{\rm H})}{(a_0a_1-k_0k_1)[m\,[(a_0-a_1)^2+4k_0k_1]-2\gamma^2(a_0+a_1)]}, \tag{11f}$$

$$\langle X_{\rm C}\dot{X}_{\rm H}\rangle = k_{\rm B}\frac{2\gamma(k_1T_{\rm C}-k_0T_{\rm H})}{m\,[(a_0-a_1)^2+4k_0k_1]-2\gamma^2(a_0+a_1)}. \tag{11g}$$

This generalizes the results given in [5] to the case of two independent trap stiffness and coupling strengths. For the case considered here, $a_0=-(\kappa+\kappa_{\rm C})$, $k_0=\kappa_{\rm C}$, $a_1=-(\kappa+\kappa_{\rm H})$, $k_1=\kappa_{\rm H}$,

$$\langle \dot{X}_{\rm C}^2\rangle = k_{\rm B}\frac{\kappa_{\rm C}(\kappa_{\rm C}T_{\rm H}-\kappa_{\rm H}T_{\rm C})}{(m/2)(\kappa_{\rm C}+\kappa_{\rm H})^2+\gamma^2(2\kappa+\kappa_{\rm C}+\kappa_{\rm H})}+\frac{k_{\rm B}T_{\rm C}}{m}. \tag{12}$$

Hence, we find substituting (12) in Eq. (5)

$$\langle \dot{Q}_{\rm C}\rangle = k_{\rm B}\frac{\kappa_{\rm C}(\kappa_{\rm C}T_{\rm H}-\kappa_{\rm H}T_{\rm C})}{(1/2)(m/\gamma)(\kappa_{\rm C}+\kappa_{\rm H})^2+\gamma(2\kappa+\kappa_{\rm C}+\kappa_{\rm H})}, \tag{13}$$

which coincides with Eq. (3) in the Main Text. The expression (4) in the Main Text for the rate of heat dissipation to the hot bath can be found following analogous steps.

B. Entropy production rate

The corresponding total entropy production rate, is defined by [7]

$$\langle \dot{S}_{\rm tot}\rangle = \langle \dot{Q}_{\rm H}/T_{\rm H}\rangle + \langle \dot{Q}_{\rm C}/T_{\rm C}\rangle + \langle \dot{S}_{\rm sh}\rangle. \tag{14}$$

The last term denotes the rate of change of the Shannon entropy $\langle S_{\rm sh}\rangle = k_{\rm B}\langle -\ln\rho_4(X_{\rm C},X_{\rm H},\dot{X}_{\rm C},\dot{X}_{\rm H})\rangle$ of the joint probability density function. This term is constant in the steady state, thus, $\langle \dot{S}_{\rm sh}\rangle=0$. In the present case, using (3) and (4) from the Main Text, and $\langle \dot{S}_{\rm sh}\rangle=0$ and Eq. (14), we find

$$\langle \dot{S}_{\rm tot}\rangle = \frac{k_{\rm B}}{T_{\rm C}T_{\rm H}}\frac{(\kappa_{\rm C}T_{\rm H}-\kappa_{\rm H}T_{\rm C})^2}{\frac{m}{\gamma}(\kappa_{\rm C}+\kappa_{\rm H})^2/2+\gamma(\kappa_{\rm C}+\kappa_{\rm H}+2\kappa)}\geq 0, \tag{15}$$

which is also given in (5) in the Main Text. As expected, the total entropy production rate of the process is always greater or equal than zero. We further note that the rates of entropy production and heat dissipated have contributions from the inertial terms (i.e., it depends on m). Figure 3b in the Main Text shows the rate of entropy production from Eq. (15) and from the MD simulations, with the latter given by the heat flows extracted by the thermostat divided by the respective temperatures.

III. DETAILED BALANCE AT THE "PSEUDO EQUILIBRIUM" POINT

As described in the Main Text, we find that at $\kappa_{\rm C}T_{\rm H} = \kappa_{\rm H}T_{\rm C}$ the average values of the heat flows and the entropy production rate all vanish. To further investigate this "pseudo equilibrium" point, we check whether in this point the Langevin Eq. (8) fulfills detailed balance, meaning that all probability flows vanish. Detailed balance is a fundamental law defining of systems in thermal equilibrium. We employ a reasoning inspired by the arguments used in [8].

To this end, we consider the flow of the 4-point joint probability density function (pdf), $\rho_4(\mathbf{x},\mathbf{v},t)$, of $\mathbf{x} = (x_{\rm C}, x_{\rm H})^{\sf T}$ and $\mathbf{v} = (\dot{x}_{\rm C}, \dot{x}_{\rm H})^{\sf T}$, appearing in the corresponding multivariate Fokker-Planck equation. The Fokker-Planck equation reads

$$\partial_t \rho_4(\mathbf{x},\mathbf{v},t) = -\nabla_x \underbrace{[\mathbf{v}\rho_4(\mathbf{x},\mathbf{v},t)]}_{=\mathbf{J}_x}, -\nabla_v \underbrace{[(\mathbf{A}\mathbf{x}-\mathbf{\Gamma}\mathbf{v})/m - \mathbf{D}_2\nabla_v]\rho_4(\mathbf{x},\mathbf{v},t)}_{=\mathbf{J}_v}, \tag{16}$$

with the probability currents $\mathbf{J}_v, \mathbf{J}_x$ and the diffusion, friction and coupling matrices defined in (8). In general, the latter are constant in steady states, and zero in equilibrium. Now we use the identity $\partial_z \rho = [\partial_z \ln(\rho)]\rho$, and define the four-dimensional phase space velocity $\mathbf{u}$ to rewrite the Fokker-Planck equation (16) as

$$\partial_t \rho_4 = -\nabla\left[\mathbf{u}\rho_4\right], \quad \text{with} \quad \mathbf{u} = (\mathbf{v}, \mathbf{u}_v)^{\sf T}, \quad \mathbf{u}_v = \mathbf{A}/m\,\mathbf{x} - \mathbf{\Gamma}/m\,\mathbf{v} - \mathbf{D}_2\nabla_v \ln\rho_4(\mathbf{x},\mathbf{v}). \tag{17}$$

The phase space velocity is connected to the probability current by $\mathbf{J} = \mathbf{u}\rho_4$. Detailed balance is fulfilled if all probability flows vanish. Hence, it is fulfilled if all components of $\mathbf{u}$ vanish. From (17) we, in turn, find that the phase space velocity vanishes if $\mathbf{D}_2^{-1}(\mathbf{A}\mathbf{x} - \mathbf{\Gamma}\mathbf{v}) = m\nabla_v \ln\rho_4$. Thus, $\mathbf{D}_2^{-1}(\mathbf{A}\mathbf{x} - \mathbf{\Gamma}\mathbf{v})$ must be the gradient of a scalar function. The latter is true if and only if

$$\nabla \times [\mathbf{D}_2^{-1}(\mathbf{A}\mathbf{x} - \mathbf{\Gamma}\mathbf{v})] = 0. \tag{18}$$

Now, since in our model, $\mathbf{A}$, $\mathbf{\Gamma}$, and $\mathbf{D}_2$ are independent of $\mathbf{x}$ and $\mathbf{\Gamma}$ and $\mathbf{D}_2$ are diagonal, the last condition is only fulfilled if $\nabla \times \mathbf{D}_2^{-1}\mathbf{A}\mathbf{x} = 0$. Concretely, inserting the coupling and diffusion matrix defined in (8) and below, this can only be satisfied, if

$$\kappa_{\rm C}T_{\rm H} = \kappa_{\rm H}T_{\rm C}. \tag{19}$$

In summary, this reasoning has revealed that the detailed balance is fulfilled if and only if (19), which coincides with the "equilibrium condition" found from the entropy production rate. We stress that this condition is irrespective of the friction coefficients, the trap stiffness and the mass of the nanoparticles, and has the same form when starting with the overdamped limit of the Langevin equations [9].

IV. COP OF THE HEAT PUMP

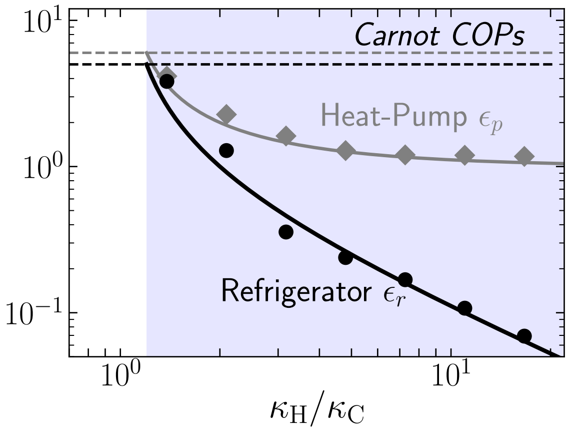

Supplementary Fig. S3: Coefficient of performance (COP) characterizing the mean efficiency of the refrigeration (black disks and line) and of the heat-pumping (grey diamonds and grey line) achieved by the nonreciprocally coupled nanoparticle system. Symbols stem from MD simulations, while the lines show the analytical predictions ($\langle\epsilon_p\rangle$ given in (20), ϵ_r given in the Main Text). Note the logarithmic axes. The horizontal dashed lines indicate the Carnot COPs, which in this case are 5 for the refrigerator and 6 for the heat pump. The Carnot values are reached in the limit $\kappa_\mathrm{H} \to \kappa_\mathrm{C}$. In this limit, the rate of extracted and pumped heat vanishes, however, vanishes, such that no heat is transferred in finite time.

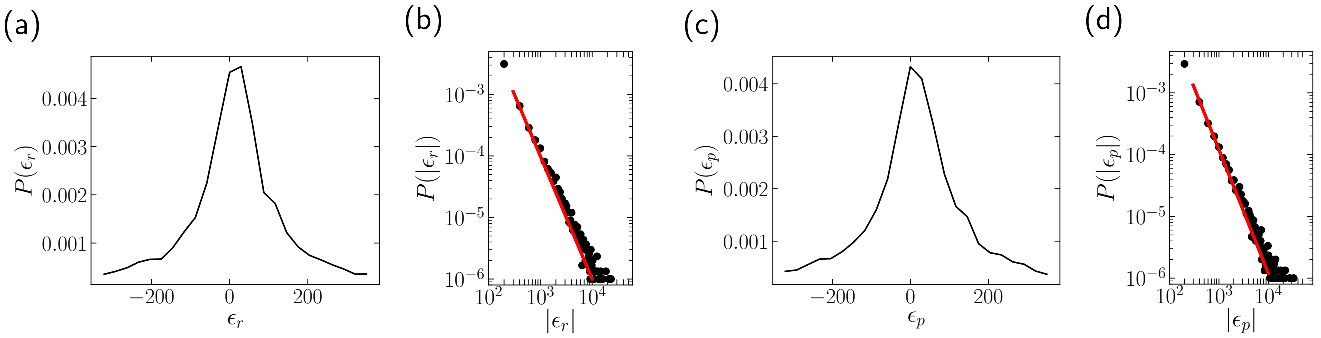

Supplementary Fig. S4: Fluctuations of the COP. (a,b) Fluctuations of the COP for the refrigerator mode, $\epsilon_r = \frac{|\dot{Q}_\mathrm{C}|}{\dot{W}}$, in linear (a) and logarithmic scale (b). (c,d) Fluctuations of the COP for the heat pump mode, $\epsilon_p = \frac{|\dot{Q}_\mathrm{H}|}{\dot{W}}$, in linear (c) and logarithmic scale (d). Note that the linear plots in (a,b) appears much more noisy, as few data points lie in the shown range, which is a consequence of the power-law distribution. The red lines in (c,d) depict power laws with exponent -2. The exponent of -2 is in good agreement with the COP of heat pump and of the refrigerator for all values of $\kappa_\mathrm{H}/\kappa_\mathrm{C}$ considered.

In the Main Text, we consider the system as a nano refrigerator, i.e., we quantify the efficiency of cooling. However, one can also use this machine as a heat pump to heat up the hot bath. The efficiency of heat pumping can be measured by an analogous coefficient of performance defined as

$$\langle\epsilon_p\rangle = \frac{|\langle\dot{Q}_\mathrm{H}\rangle|}{\langle\dot{W}\rangle} = \frac{|\langle\dot{Q}_\mathrm{H}\rangle|}{\langle\dot{Q}_\mathrm{H}\rangle + \langle\dot{Q}_\mathrm{C}\rangle} = \frac{\kappa_\mathrm{H}}{\kappa_\mathrm{H} - \kappa_\mathrm{C}}. \tag{20}$$

Like the COP of the refrigerator mode, the COP of the heat pump is bounded by the Carnot value $\langle\epsilon_p\rangle \leq \frac{T_\mathrm{H}}{T_\mathrm{H}-T_\mathrm{C}}$. Supplementary Fig. S3 displays both COPs for the refrigerator and heat pump, from simulations and from the theoretical predictions. Supplementary Fig. S4 shows the distributions of the fluctuating COPs, which are discussed in the Main Text.

V. DERIVATION OF THE DISTRIBUTION OF POWER [EQ. (9) IN THE MAIN TEXT]

Here, we derive the distribution of the instantaneous (stochastic) power. We make use of the explicit expression for the stationary joint probability density for the position and velocities of the two nanoparticles $\rho_4(x_{\rm C}, x_{\rm H}, v_{\rm C}, v_{\rm H})$, which is a four-variate Gaussian distribution with zero mean and covariance matrix $\mathbf{C}$ (7) as given in this supplemental in Sec. II.

To access the distribution of $\dot{W}_j$ with $j \in \{{\rm C}, {\rm H}\}$, we first recall that

$$\dot{W}_j = ({\rm d}t)^{-1}(\kappa_j x_l \circ {\rm d}x_j) = \kappa_j x_l \circ v_j, \tag{21}$$

with $l \neq j$ which we assume throughout this section. From ρ_4, we can derive $P(\dot{W}_j)$ by the following integral

$$P(\dot{W}_j) = \int_{-\infty}^{\infty} {\rm d}x_j \int_{-\infty}^{\infty} {\rm d}x_l \int_{-\infty}^{\infty} {\rm d}v_j \int_{-\infty}^{\infty} {\rm d}v_l \, \rho_4(x_j, x_l, v_j, v_l)\delta(\dot{W}_j - \kappa_j x_l v_j) \tag{22}$$

$$= \int_{-\infty}^{\infty} {\rm d}x_l \int_{-\infty}^{\infty} {\rm d}v_j \, \rho_2(x_l, v_j)\delta(\dot{W}_j - \kappa_j x_l v_j). \tag{23}$$

Here,

$$\rho_2(x_l, v_j) = \int_{-\infty}^{\infty} {\rm d}x_j \int_{-\infty}^{\infty} {\rm d}v_l \rho_4(x_j, x_l, v_j, v_l) = \mathcal{N}_j^{-1} \exp(-\alpha_j x_l^2 + \beta_j x_l v_j - \zeta_j v_j^2), \tag{24}$$

is the joint stationary distribution of the position of the $l-$nanoparticle and the velocity of the $j-$nanoparticle, with $j \neq l$. It is given by a bivariate normal distribution with normalization constant $\mathcal{N}_j$, and coefficients

$$\alpha_j = \frac{1}{2\langle X_l^2\rangle(1-\psi_j^2)}, \quad \beta_j = \frac{\psi_j}{\sqrt{\langle X_l^2\rangle\langle \dot{X}_j^2\rangle(1-\psi_j^2)}}, \quad \zeta_j = \frac{1}{2\langle \dot{X}_j^2\rangle(1-\psi_j^2)}, \quad \psi_j = \frac{\langle X_l \dot{X}_j\rangle}{\sqrt{\langle X_l^2\rangle\langle \dot{X}_j^2\rangle}}, \tag{25}$$

which are functions of the covariances of the position and velocities of the nanoparticles. Therefore, the distribution of the power depends on the functions $\alpha_j, \beta_j, \zeta_j$ defined in (25), which in turn depend on $\langle X_l^2\rangle$, $\langle X_l \dot{X}_j\rangle$ and $\langle \dot{X}_j^2\rangle$. See Sec. II A of this supplemental for explicit analytical expressions of the covariances in terms of the physical parameters of the system.

Using the explicit expression of ρ_2 given by Eq. (24) and integrating out the delta distribution in Eq. (23), we obtain

$$P(\dot{W}_j) = \int_{-\infty}^{\infty} {\rm d}x_l \int_{-\infty}^{\infty} {\rm d}v_j \rho_2(x_l, v_j) \, \delta\left(\frac{\dot{W}_j}{\kappa_j x_l} - v_j\right) = \int_{-\infty}^{\infty} {\rm d}x_l \rho_2\left(x_l, \frac{\dot{W}_j}{\kappa_j x_l}\right) \tag{26}$$

$$= \frac{1}{\mathcal{N}_j}\int_{-\infty}^{\infty} {\rm d}x_l \exp\left(-\alpha_j x_l^2 + \beta_j x_l \frac{\dot{W}_j}{\kappa_j x_l} - \zeta_j \frac{\dot{W}_j^2}{\kappa_j^2 x_l^2}\right) = \frac{1}{\mathcal{N}_j} \exp\left(\frac{\beta_j}{\kappa_j}\dot{W}_j\right)\int_{-\infty}^{\infty} {\rm d}x_l \exp\left(-\alpha_j x_l^2 - \frac{\zeta_j \dot{W}_j^2}{\kappa_j^2}\frac{1}{x_l^2}\right). \tag{27}$$

Changing variables $y = \sqrt{\alpha_j} x_l$ and absorbing the Jacobian of the transformation in the new normalization constant $\mathcal{Z}_j = \alpha_j^2 \mathcal{N}_j$ yields

$$P(\dot{W}_j) = \frac{1}{\mathcal{N}_j} \exp\left(\frac{\beta_j}{\kappa_j}\dot{W}_j\right)\int_{-\infty}^{\infty} \frac{{\rm d}y}{\alpha_j^2} \exp\left(-y^2 - \frac{\zeta_j \dot{W}_j^2}{\kappa_j^2}\frac{\alpha_j}{y^2}\right) = \frac{1}{\mathcal{Z}_j} \exp\left(\frac{\beta_j}{\kappa_j}\dot{W}_j\right)\int_{-\infty}^{\infty} {\rm d}y \exp\left(-y^2 - \frac{b}{y^2}\right). \tag{28}$$

with $b \equiv (\zeta_j \alpha_j \dot{W}_j^2)/\kappa_j^2$. Applying the property $\int_{-\infty}^{\infty} {\rm d}y \exp\left(-y^2 - \frac{b}{y^2}\right) = \sqrt{\pi}\exp(-2\sqrt{b})$ which holds for all $d > 0$, Eq. (28) yields the distribution of the power exerted on the $j \in \{{\rm C}, {\rm H}\}$ nanoparticle

$$P(\dot{W}_j) = \frac{1}{\mathcal{Z}_j} \exp\left[\frac{\beta_j}{\kappa_j}\dot{W}_j - 2\frac{\sqrt{\zeta\alpha_j}}{|\kappa_j|}|\dot{W}_j|\right], \tag{29}$$

which coincides with Eq. (9) in the Main Text.

From the distributions (29), we can further deduce analytical expressions for any moment of the power. In particular, the variance of the power reads:

$$
\begin{aligned}
\mathrm{Var}(\dot{W}_j) &= \left(\int_{-\infty}^{\infty} P(\dot{W}_j)\dot{W}_j\right)^2 - \int_{-\infty}^{\infty} P(\dot{W}_j)\dot{W}_j^2 \\
&= \frac{1}{\mathcal{Z}}\left[\frac{2\kappa_j^3}{(2\sqrt{\alpha_j\zeta_j}+\beta_j)^3} + \frac{2\kappa_j^3}{(2\sqrt{\alpha_j\zeta_j}-\beta_j)^3} - \left(\frac{\kappa_j^2}{(2\sqrt{\alpha_j\zeta_j}+\beta_j)^2} + \frac{2\kappa_j^2}{(2\sqrt{\alpha_j\zeta_j}-\beta_j)^2}\right)^2\right].
\end{aligned}
\tag{30}
$$

Supplementary Fig. S5 depicts the mean and the variance of the power fluctuations. We find good qualitative agreement between theory [Eq. (30)] and the MD simulation results.

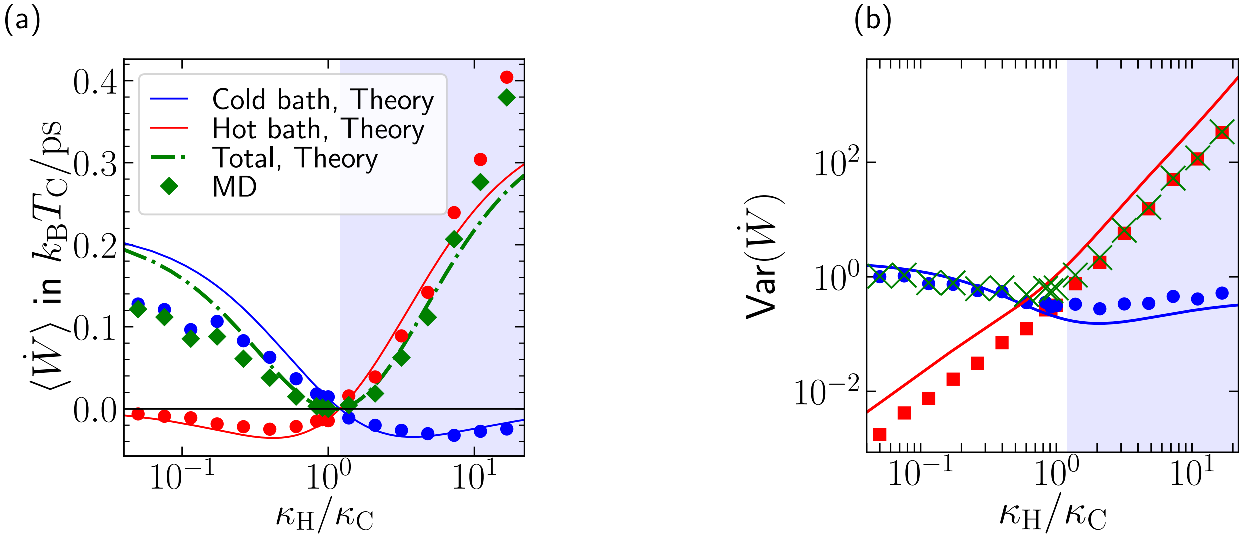

Supplementary Fig. S5: Mean value and variance of the the power exerted on the nanoparticles. (a) Mean value, (b) variance; for the cold particle ($\dot{W}_C$, blue), the hot particle ($\dot{W}_H$, red) and the total power ($\dot{W} = \dot{W}_C + \dot{W}_H$, green). The values obtained from MD simulations are shown as symbols, the theoretical prediction are shown as lines.

VI. STABILITY CONDITIONS

Here we study the stability of the process described by the Langevin equation given in Eq. (1) in the Main Text, using the matrix equation given in (8). The stability conditions can be found from the roots of the eigenvalues of the generalised coupling matrix $-\mathbf{B}$. The eigenvalues read

$$\lambda_{1/2} = \frac{1}{2m}\left(-\gamma \pm \sqrt{\gamma^2 - 4m\kappa}\right), \tag{31}$$

$$\lambda_{3/4} = \frac{1}{2m}\left(-\gamma \pm \sqrt{\gamma^2 - 4m(\kappa + \kappa_H + \kappa_C)}\right). \tag{32}$$

The two largest eigenvalues are those with the respective plus sign. From them, we find the stability conditions $-\gamma + \sqrt{\gamma^2 - 4m\kappa} < 0$, thus $\kappa > 0$, and $-\gamma + \sqrt{\gamma^2 - 4m(\kappa + \kappa_H + \kappa_C)} < 0$, thus $\kappa_H + \kappa_C > -\kappa$. To summarize, the system is stable if

$$\kappa > 0, \text{ and } \kappa_H + \kappa_C > -\kappa. \tag{33}$$

We note that these conditions are independent of m and coincide with the stability boundaries of the overdamped system (as expected). Further note that the eigenvalues become complex if $\gamma^2 < 4m\kappa$, or $\gamma^2 < 4m(\kappa + \kappa_C + \kappa_H)$. For the parameter choices considered in the MD simulations, all eigenvalues have imaginary parts, indicating oscillatory behaviour (for all considered κ_H, κ_C values). The oscillatory character of the stochastic dynamics (which stems from

the inertial terms) manifests itself in the negative values in the velocity autocorrelation function (see Supplementary Fig. S2).

[1] M. C. Wang and G. E. Uhlenbeck, Reviews of modern physics **17**, 323 (1945).
[2] K. Sekimoto, *Stochastic energetics*, vol. 799 (Springer, 2010).
[3] S. A. M. Loos and S. H. L. Klapp, Sci. Rep. **9**, 2491 (2019).
[4] C. Kwon, P. Ao, and D. J. Thouless, Proceedings of the National Academy of Sciences **102**, 13029 (2005).
[5] Y. Bae, S. Lee, J. Kim, and H. Jeong, Physical Review E **103**, 032148 (2021).
[6] C. Kwon, J. D. Noh, and H. Park, Physical Review E **83**, 061145 (2011).
[7] U. Seifert, Rep. Prog. Phys. **75**, 126001 (2012).
[8] J. B. Weiss, Tellus A: Dynamic Meteorology and Oceanography **55**, 208 (2003).
[9] S. A. M. Loos and S. H. L. Klapp, New Journal of Physics **22**, 123051 (2020).
